# Transient Terahertz Oscillations During Photoinduced Polarization Topology Reconfiguration in Ferroelectric Superlattices

**DOI:** 10.1002/advs.202522387

**Published:** 2026-02-03

**Authors:** Deepankar Sri Gyan, Hyeon Jun Lee, Xiangwei Guo, Youngjun Ahn, Samuel D. Marks, Mohammed H. Yusuf, Matthew Dawber, James M. Glownia, Diling Zhu, Takahiro Sato, Sanghoon Song, Haidan Wen, Jia‐Mian Hu, Paul G. Evans

**Affiliations:** ^1^ Department of Materials Science and Engineering University of Wisconsin‐Madison Madison Wisconsin USA; ^2^ Department of Materials Science and Engineering Kangwon National University Samcheok South Korea; ^3^ Materials Science Division Argonne National Laboratory Lemont Illinois USA; ^4^ Department of Physics and Astronomy Stony Brook University Stony Brook New York USA; ^5^ Department of Physics University of Vermont Burlington Vermont USA; ^6^ Linac Coherent Light Source SLAC National Accelerator Laboratory Menlo Park California USA; ^7^ X‐ray Science Division Argonne National Laboratory Lemont Illinois USA

**Keywords:** ferroelectric topologies, free‐electron laser radiation, THz materials, THz polarization dynamics, time‐resolved x‐ray diffraction

## Abstract

Terahertz resonances embedded in crystalline heterostructures could close a spectral gap between conventional electronics and photonics while opening new windows on phenomena in non‐equilibrium lattice dynamics. We show that femtosecond optical screening of the depolarization field in epitaxial PbTiO_3_/SrTiO_3_ superlattices launches a collective polar mode that oscillates near 1 THz and coherently spans the entire mini‐Brillouin zone. Wave‐vector‐resolved pump–probe X‐ray diffraction resolves a nearly dispersion‐less oscillation at 0.87 and 0.94 THz at the zone boundary and zone center, respectively, persisting for ∼2.5 ps, corresponding to a weakly damped resonance. Dynamical phase‐field simulations reveal the origin of the mode to mesoscopic rotation of closure‐domain textures during the photoexcited transition from an unscreened to a screened electrostatic state. Varying the PbTiO_3_ and SrTiO_3_ ratio tunes the mode frequency continuously from 0.9 to 1.4 THz, providing a quantitative design rule for frequency‐selectable THz oscillators in ferroelectric heterostructures. By coupling nanoscale polarization reconfiguration to long‐wavelength coherent dynamics, this work establishes depolarization‐field engineering to topology‐driven THz functionality and expanding the landscape of collective lattice dynamics.

## Graphical abstract

Terahertz resonances in crystalline heterostructures could close a spectral gap between conventional electronics and photonics while opening new windows on non‐equilibrium lattice dynamics. Femtosecond optical screening of the depolarization field in epitaxial PbTiO_3_/SrTiO_3_ superlattices launches a collective polar mode that oscillates near 1 THz and coherently spans the entire mini‐Brillouin zone and appears in ultrafast x‐ray diffraction measurements.

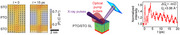

## 1 Introduction

Terahertz (THz) frequencies occupy the spectral gap between electronics and photonics, bridging microwave electronics on the low‐frequency side and infrared/optical photonics at higher frequencies. The characteristic timescales of THz phenomena (sub‐picosecond) and energies (few meV) match a broad class of low‐energy degrees of freedom in solids, such as collective excitations in quantum materials, as well as resonant processes relevant to sub‐THz wireless communications [1, 2]. Consequently, the ability to control electromagnetic responses in this regime opens opportunities across condensed‐matter physics and engineering. However, there is a persistent challenge in developing structures with specific and selectable electromagnetic properties in the THz frequency regime for numerous applications in electronics, optics, and communications [3, 4]. The design challenge for these materials can be briefly stated as a need to develop systems with resonances in the THz frequency regime that are strongly coupled to electromagnetic radiation. Creative approaches include metamaterial engineering through layering, patterning of features to induce vortex formation at domain boundaries in ferroic systems, harnessing size effects in ferroelectric nanoparticles, and leveraging optically induced states [5, 6, 7, 8, 9]. These approaches have the advantage of allowing advanced design strategies to explore a large and challenging parameter space [5], but are frequently limited by a small range of available spontaneously formed patterns, by the challenges arising from lithography, or the limited range of properties possessed by homogeneous materials. Materials design of crystalline heterostructures with intrinsic THz resonances has the potential to resolve these challenges by using compositional tunability.

Epitaxial ferroelectric/dielectric (FE/DE) superlattices (SLs) feature nanoscale ferroelectric polarization configurations resulting from the relationships among the depolarizing field due to electrical boundary conditions, epitaxial strain, and composition. The resulting complex spatial distributions and topologies have collective dynamics in the THz regime [10, 11, 12, 13, 14, 15]. Because the characteristic THz frequencies strongly depend on the electro‐elastic restoration forces acting on mesoscopic polarization configurations, FE/DE SLs can provide THz frequency tunability [15]. THz‐frequency collective modes in oxide superlattices can be resonantly driven with tailored THz pump pulses or excited via modes generated via acoustic‐phonon excitation [16, 17, 18]. In contrast, our work focuses on optical or electrical excitation, which perturbs the polarization domains and, through transient screening of the depolarizing fields, generates non‐equilibrium THz collective modes that assist dynamic domain transformations in the superlattice.

We show here that photoexcited screening of depolarizing fields in PbTiO_3_/SrTiO_3_ SLs launches a transient collective oscillation of mesoscopic polar textures whose frequency resides near 1 THz. The dynamics of the polarization screening‐driven transition are lightly damped and exhibit a lifetime corresponding to the duration of the transition between the equilibrium configuration and the optically excited intermediate state, a few periods of the frequency characteristic of the oscillation of the polarization configuration. Wavevector‐resolved time‐domain measurement employing femtosecond free‐electron X‐ray diffraction reveals a nearly dispersionless mode spanning the mini‐Brillouin zone. Complementary dynamical phase‐field simulations link the oscillations to polarization rotation at domain boundaries and provide a framework for materials design. THz oscillation modes can be tuned by adjusting the FE and DE layer fraction, a quantitative design rule for THz resonances embedded in FE/DE SL heterostructures.

**FIGURE 1:**
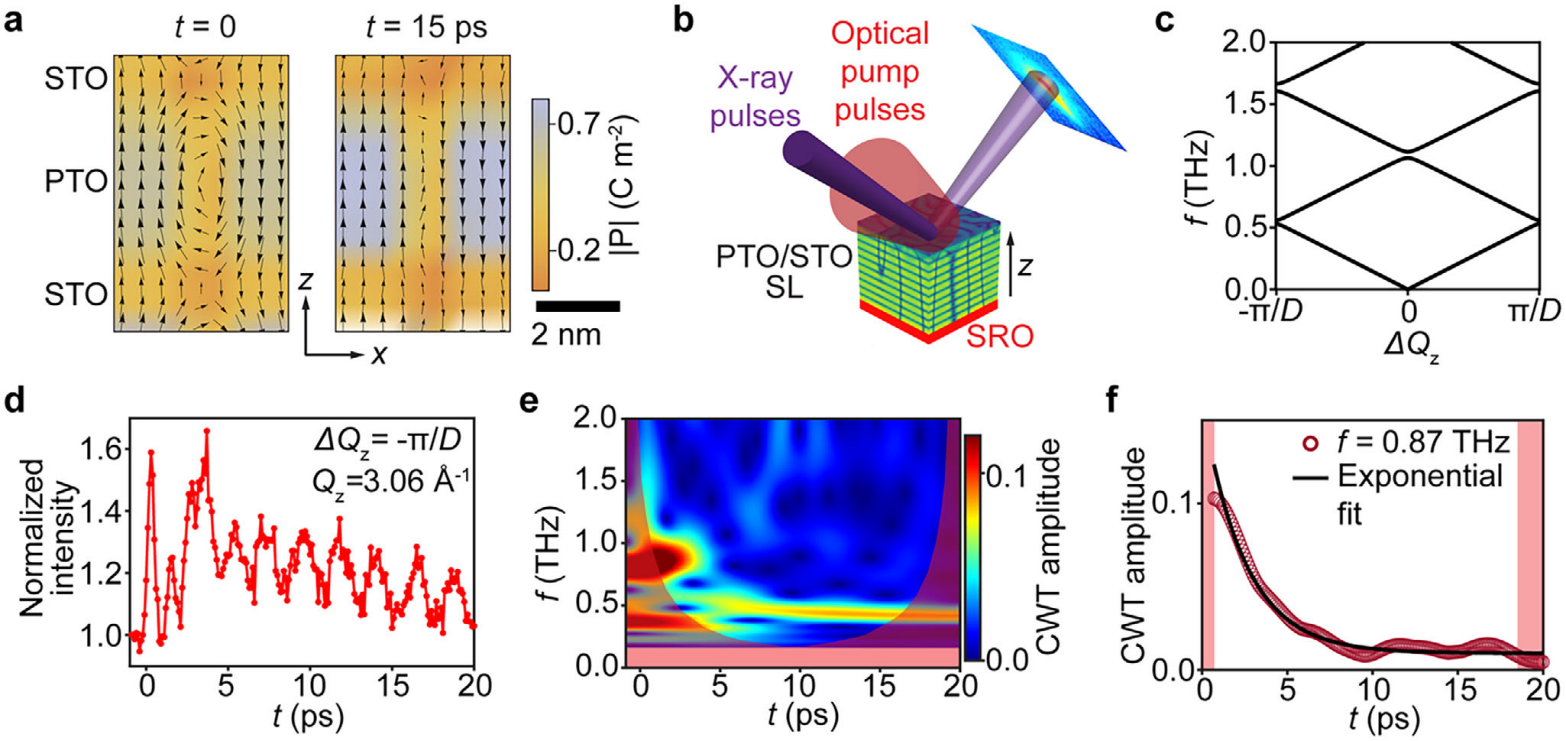
Excitation and detection of THz ferroelectric polarization dynamics in ferroelectric/dielectric superlattices. (a) Polarization configuration in (PTO)_8_/(STO)_3_ superlattice (SL), computed using dynamical phase‐field simulations. Configurations are shown (left) under equilibrium conditions before optical excitation at *t* = 0 and (right) at *t* = 15 ps after reaching a steady state following a step change in the dielectric permittivity of the STO layer that approximates the optically induced screening of the depolarization field. (b) Schematic of the ultrafast optical pump/free‐electron‐laser X‐ray diffraction probe experiment. (c) Frequency‐wavevector dispersion for longitudinal acoustic phonon modes predicted by an elastic continuum model of the SL, spanning one mini‐BZ. (d) Time dependence of the diffracted X‐ray intensity at the wavevector corresponding to the mini‐BZ boundary, *Q*_z_ = 3.06 Å^−1^. (e) *f*—*t* map of intensity profile in Figure 1d obtained using continuous wavelet transform (CWT) analysis. The shaded region corresponds to the part of the map outside the boundary of the cone‐of‐influence (COI) for the CWT. (f) Time dependence of the CWT amplitude at *f =* 0.87 THz (points). The shaded regions correspond to times for which the COI indicates that simultaneous reporting of values of *f* and *t* are unreliable. The fit of an exponential decay (line) does not include the points outside the COI.

The equilibrium configuration of polarization obtained from a dynamical phase‐field simulation of a SL with a repeating unit consisting of 8 unit cells (u. c.) of the FE oxide PbTiO_3_ (PTO) and 3 u. c. of the DE oxide SrTiO_3_ (STO), a total repeating unit thickness *D* = 4.2 nm, is shown in Figure 1a. The configuration consists of a nanodomain pattern characterized by (i) a reduction of the PTO polarization by 25% with respect to its equilibrium bulk value and (ii) a non‐zero polarization of the STO component. The simulated polarization configuration also includes a flux closure texture near domain walls, featuring an in‐plane component of the polarization in the PTO and STO components. In experiments, optical excitation results in the generation of a large population of mobile charge carriers in the (PTO)_8_/(STO)_3_ SL, which screens the depolarization field, an effect also widely observed in other ferroelectrics, including BiFeO_3_ [19, 20]. The screening is introduced in the simulation using a change in the relative dielectric permittivity of the STO layer from its equilibrium value, 40, to a far larger aribtrarily selected value, 10^4^. The dynamical phase‐field model shows that screening leads to polarization rotation near the domain walls, resulting in the final configuration also shown in Figure 1a for simulation at 15 ps, a time at which the transient dynamics following optical excitation are complete. The PTO component of the simulated screened configuration has a 17% larger out‐of‐plane polarization than in the unscreened configuration [21].

Changes in polarization are accompanied by structural distortion that can be readily probed using X‐ray diffraction. The reciprocal space of the SL along the out‐of‐plane *z* direction is described using wavevector *Q*_z_, with a series of X‐ray reflections arising from the SL periodicity appearing around each Bragg reflection of the SL. These SL reflections are separated by Δ*Q*_z_ = 2π/*D*. The mini‐Brillouin zone (BZ) of the SL extends over a range Δ*Q*_z_ from ‐π/*D* to π/*D*. The SL reflections are labeled using an integer *l*, with *l* = 0 at each Bragg reflection and positive and negative *l* indicating reflections at greater and lower values of the *Q*_z_ than the SL reflection, respectively. In the equilibrium state, before photoexcitation, the polarization configuration is a nanodomain pattern with an in‐plane period of 8.3 nm, as measured using X‐ray diffuse scattering [19]. The domain pattern has a coherence length of several domain periods, with domains that extend vertically through approximately the entire extent of the SL thin film [22].

The excitation of the FE/DE SL following optical excitation was experimentally probed using ultrafast free‐electron laser (FEL) X‐ray diffraction with a photon energy of 9.5 keV, as in Figure 1b. The measurements were conducted at 120°C and compared with room‐temperature measurements to evaluate possible charge‐trapping effects [23]. Unlike previous studies at room temperature [19, 23], in which photoexcited carriers can stabilize a monodomain configuration, our measurements do not probe a regime of complete domain switching. The persistence of the domain pattern during optical excitation allows us to focus on the ultrafast polarization dynamics immediately following optical excitation, prior to any long‐timescale saturation or defect‐mediated trapping effects. Pump probe measurements employed a 50‐fs duration optical pump pulse with a wavelength of 400 nm. Structural changes in the SL following excitation led to a temporal variation of the diffracted X‐ray intensity. The SL polarization was probed using a wavevector‐resolved time‐domain measurement of the diffracted X‐ray intensity as a function of *Q*_z_ and time *t* relative to the optical excitation. Structural changes with wavevector *Q*_z_ cause X‐ray intensity changes at the corresponding wavevector in reciprocal space [24]. The measurements consisted of time and wavevector mappings of the scattering X‐ray intensity spanning the mini‐BZ, for example, from one SL reflection (e.g., *l =* 0) to an adjacent SL reflection (e.g., *l* = ‐1).

A key issue in interpreting the results of the time‐domain measurements is that there is a corresponding frequency‐domain interpretation of results that can be accessed by determining the oscillation frequencies of the time‐domain response to transient excitation. Comparing the frequencies of these measurements with theoretical and computational predictions provides a new perspective on dynamical phenomena in complex heterostructures. The diffraction approach allows the frequency‐wavevector dispersion of the oscillation to be characterized throughout the mini‐BZ. A continuous wavelet transform (CWT) analysis was used to investigate the time‐dependent frequency spectrum of the X‐ray intensity at each *Q*_z_. The CWT provides the amplitude of oscillations with characteristic frequency *f* as a function of time and has boundaries of its validity set by the uncertainty principle [25].

The frequency‐wavevector dispersion of the longitudinal acoustic phonon modes of the SL serves as a guide for identifying zone‐folding modes and for distinguishing longitudinal acoustic modes from oscillations of the polarization configuration. The longitudinal acoustic dispersion was calculated using the equilibrium properties of the SL, employing an approach described in Section S1. The predicted acoustic dispersion, Figure 1c, has a reciprocal‐space periodicity with period 2π/*D* due to zone folding [21, 26]. Gaps in the frequency‐wavevector dispersion arise at the center and boundaries of the mini‐BZ due to the acoustic mismatch between PTO and STO [26, 27].

## 2 Results and Discussion

Time‐resolved X‐ray diffraction measurements following optical excitation reveal oscillations of the diffracted intensity with frequencies in the THz regime. The diffracted X‐ray intensity measured at 120 °C for *t* = −1.5 to 20 ps at *Q*_z_ = 3.06 Å^−1^ is shown in Figure 1d. *Q*_z_ = 3.06 Å^−1^ corresponds to the mini‐BZ boundary between the SL *l* = −1 (*Q*_z_ = 2.97 Å^−1^) and SL *l* = 0 (*Q*_z_ = 3.13 Å^−1^) SL X‐ray reflections near the SL 002 Bragg reflection. The intensity in Figure 1d oscillates with a period of 1.1 ps, with an exponential decay lifetime of 2.5 ps. The oscillations with a 1.1 ps period do not appear at longer times. In addition, a longer‐lived oscillation with a period of 2.5 ps spans the entire time range in Figure 1d. The domain diffuse scattering intensity around the SL *l* = 0 reflection was recorded for pulses in which the optical intensity was off, as described in Section S8. There was no systematic variation in the diffuse scattering intensity, indicating that the superlattice re‐establishes its initial polarization texture between excitation pulses.

Figure 1e shows the *f*–*t* distribution of CWT amplitude obtained from the intensity in Figure 1d. Details of the CWT analysis are in Section S2. There are ranges of values of *f* and *t* for which separate frequency and time analysis is inaccurate due to the uncertainty principle, particularly in the regime of small *t* and low *f*. The regions of incompatibility are indicated by shading outside the cone of influence (COI), in Figure 1e [25].

The CWT amplitude map in Figure 1e exhibits a peak at *f* = 0.87 THz for *t* less than 4 ps, corresponding to the 1.1 ps period oscillation in Figure 1d. The 0.87 THz frequency does not match the longitudinal acoustic frequency predicted for mini‐BZ boundaries in Figure 1c. The predicted longitudinal acoustic phonon modes do, however, appear in the CWT in Figure 1e at a frequency of 0.48 THz and span the entire time range of the measurement. The CWT analysis further shows that the longitudinal acoustic phonon oscillations in Figure 1e are split into two frequencies with a difference equal to a frequency gap of 0.06 THz, as described in Section S3. The frequency splitting in the longitudinal acoustic phonon oscillation was 0.06 THz was measurable because the acoustic oscillations persisted for many picoseconds. The time dependence of CWT amplitude at *f* = 0.87 THz, Figure 1f, has an exponential decay of 2.5 ps corresponding to the lifetime of the *f* = 0.87 THz zone‐boundary oscillations. We hypothesize that these non‐equilibrium polarization oscillations, which do not overlap with the acoustic dispersion of the lattice, can radiate energy into acoustic modes, providing an additional damping channel and resulting in the shorter lifetimes (∼2.5 ps) of the *f* ≈ 0.87 THz zone‐boundary mode.

**FIGURE 2:**
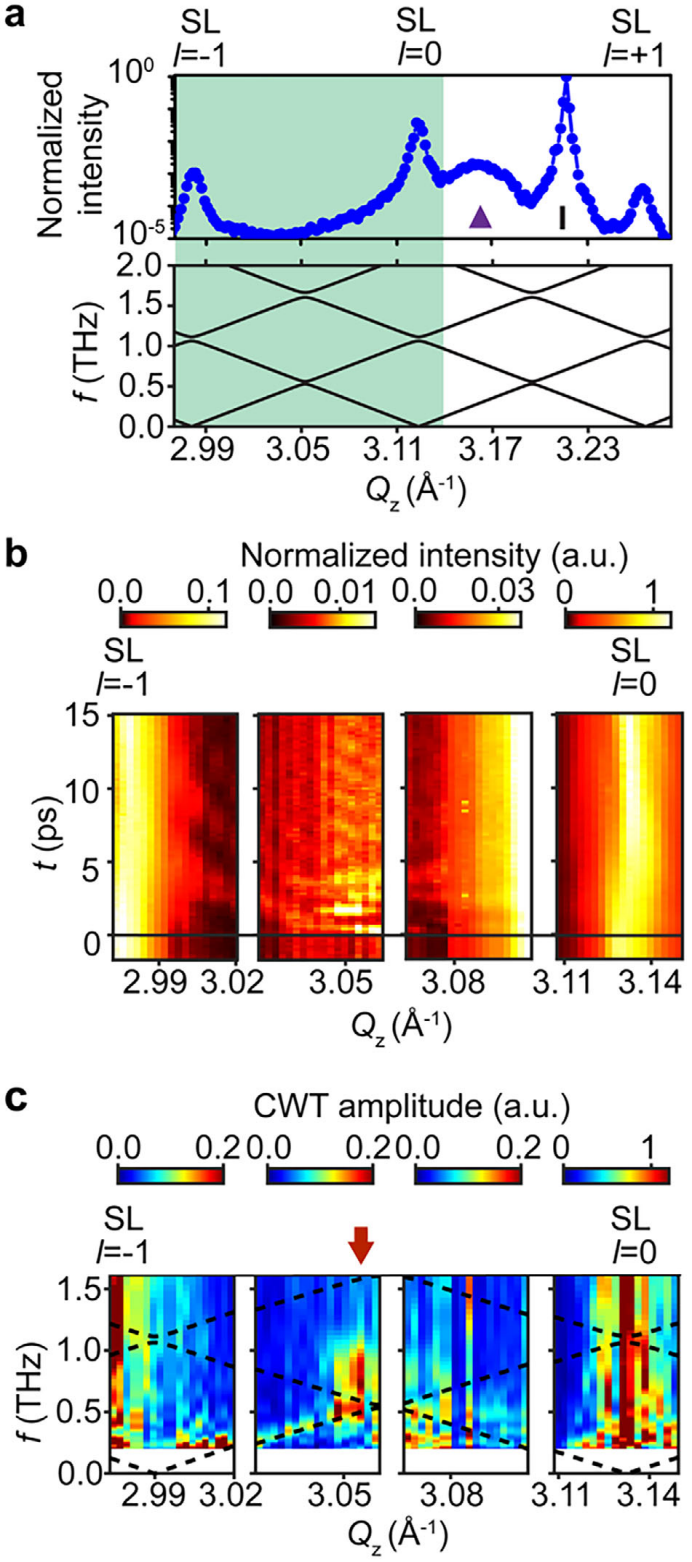
Wavevector resolved dynamics of structural and polarization dynamics. (a) Equilibrium X‐ray diffraction pattern of the SL with *l* = ‐1, 0, and 1 reflections. Intensity maxima corresponding to the STO 002 (vertical line) and SRO 002 (triangle) reflections are labeled. The bottom panel shows a repeating‐zone representation of several mini‐BZs of the predicted longitudinal acoustic phonon dispersion. (b) X‐ray intensity as a function of *Q*_z_ and *t* with a wavevector range spanning the *l* = ‐1 and *l* = 0 reflections of the SL. This region is highlighted in Figure 2a. (c) Time‐averaged CWT amplitude obtained from the intensity data in b), with a marker indicating the wavevector at which the measurement presented at Figure 1d was acquired. The dashed lines correspond to the predicted longitudinal acoustic phonon dispersion.

The dispersion of the oscillations was obtained by repeating the ultrafast diffraction measurement for a wide range of wavevectors. A map guiding the selection of wavevectors is shown in Figure 2a, along with a steady state X‐ray diffraction pattern spanning a range of reciprocal space that includes the SL *l* = −1, 0, and +1 reflections near the SL 002 Bragg reflection. The repeating‐zone representation of the longitudinal acoustic dispersion in Figure 2a indicates that SL Bragg and the satellite reflections arise at the mini‐BZ centers, as expected.

The diffracted X‐ray intensity measured at 120 °C in a *t*‐*Q*_z_ range spanning the shaded wavevectors in Figure 2a is shown in Figure 2b. Each panel in Figure 2b is plotted using an individually optimized color scale to ensure that low‐intensity scattering near the mini‐BZ boundary is visible, and because a uniform scale would overemphasize the high‐intensity scattering in the wavevector range near the Bragg reflection. A series of features arising from the generation and propagation of a longitudinal acoustic pulse is apparent in Figure 2b. First, the intensity within a narrow wavevector range |Δ*Q*_z_| ≈ 0.01 Å^−^^1^ around the SL *l* = ‐1 reflection at *Q*_z_ = 2.97 Å^−1^ exhibits low‐frequency temporal oscillations with a period longer than 5 ps. The oscillation period systematically decreases, corresponding to increased frequency, for wavevector away from the mini‐Brillouin‐zone center, consistent with the expected dispersion of longitudinal acoustic modes. In addition to these acoustic modes, high‐frequency oscillations are apparent in Figure 2b, including near *Q*_z_ = 3.06 Å^−1^, for which the intensity is shown in Figure 1d.

The wavevector dependence of the oscillations is shown in the *f*‐*Q*_z_ map in Figure 2c, obtained by computing the time average of the CWT of the *t*‐*Q*_z_ data. As expected from the inspection of the *t*‐*Q*_z_ data, there are two key phenomena apparent in Figure 2c:

- *Acoustic oscillations*. The dispersion predicted by the elastic model is plotted with dotted lines in Figure 2c. The CWT amplitude follows the given dotted lines in Figure 2c, indicating that this feature arises from the acoustic phonon dispersion. These acoustic oscillations are further discussed in Section S4.
- *Oscillations not following the acoustic dispersion*. In addition to the acoustic modes, CWT amplitude appears at *f* = 0.87 THz in a wavevector range across the entire mini‐BZ. The oscillation in this frequency range in Figure 2c is consistent with the observation at *Q*_z_ = 3.06 Å^−1^ in Figure 1d. An additional oscillation at *f* = 0.48 THz is evident in Figure 2c in the range of wavevectors between *Q*_z_ = 3.05 Å^−1^ and *Q*_z_ = 3.15 Å^−1^. The single *Q*_z_ measurement in Figure 1e shows a broad 0.4–0.5 THz feature that have distinct time dependences; the two relevant modes cannot be clearly resolved at this wavevector. In contrast, the dispersion map in Figure 2c clearly separates these two contributions: one branch follows the acoustic dispersion, whereas the second remains nearly non‐dispersive as the wavevector ranges from the mini‐BZ boundary toward the mini‐BZ center at the SL *l* = 0 reflection. The nearly non‐dispersive *f* = 0.48 THz and *f* = 0.87 THz frequency modes are not present in the predicted acoustic phonon dispersion of the SL. The non‐dispersive 0.48 THz mode in Figure 2c is distinct from the acoustic mode observed for a specific wavevector in Figure 1e. High‐frequency oscillations at the SL Bragg (*l* = 0) and *l* = ‐1 reflections in Figure 2b,c are partially obscured by the high intensity of scattering near these reflections. As discussed below, the modes at *f* = 0.48 THz and *f* = 0.87 THz are consistent with dynamics arising from polarization reorientation following optical excitation.

**FIGURE 3:**
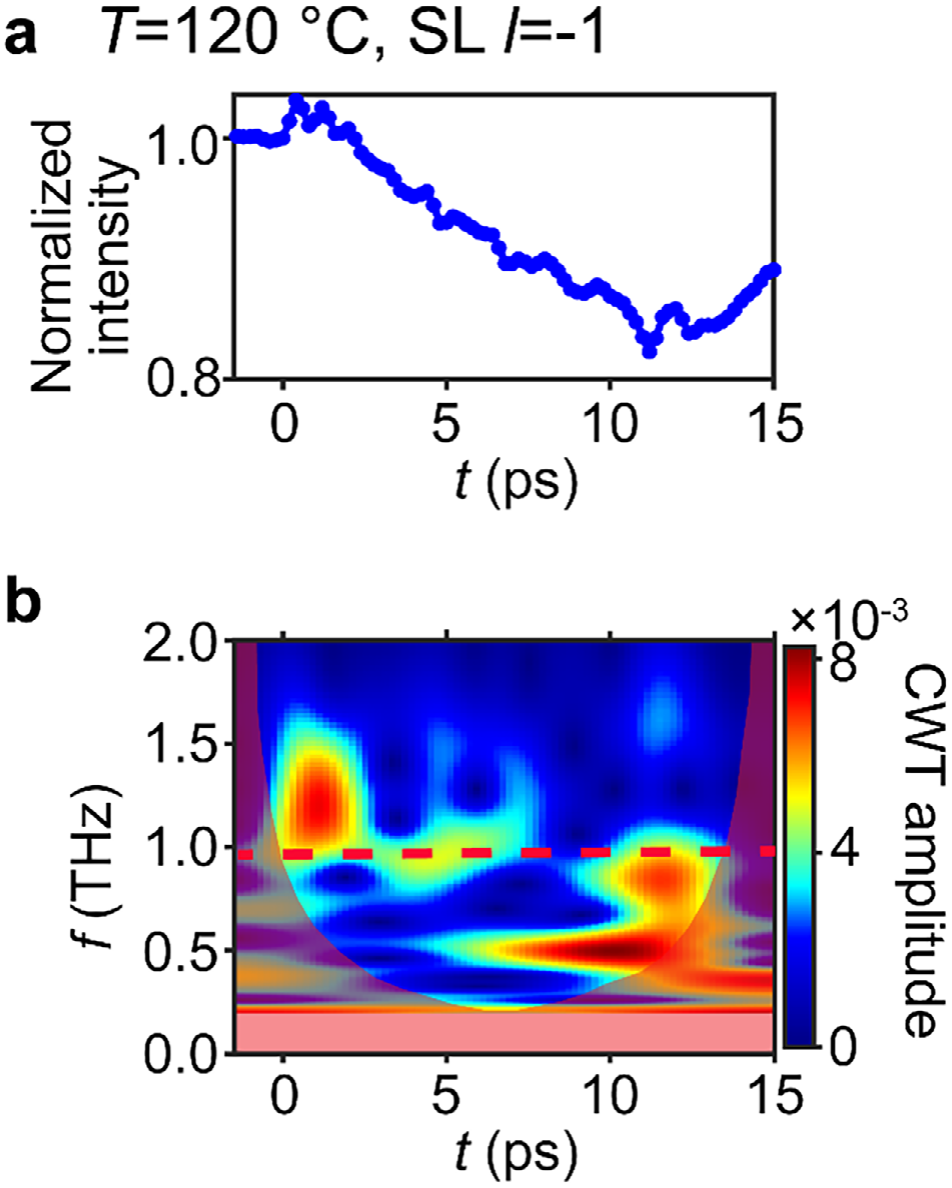
Polarization dynamics at SL *l* = ‐1 reflection. (a) X‐ray diffraction intensity of the SL *l* = ‐1 reflection as a function of time, and (b) CWT *f*‐*t* maps for the SL *l* = ‐1 reflection measured at 120 °C. The dashed line indicates the frequency of the mode associated with the optically induced polarization oscillation.

The THz‐frequency scale oscillations are also apparent, but with significantly lower amplitude, in the intensity at the center of the mini‐BZ, but with significantly lower amplitude than at the mini‐BZ boundary. Figure 3a shows the time‐dependence of the diffracted X‐ray intensity at *l* = −1, *Q*_z_ = 2.97 Å^−1^, at 120°C, normalized to before *t* = 0. Figure 3b shows *f*‐*t* maps of the CWT amplitude for the dynamics of the corresponding at the mini‐BZ center. The intensity of the SL *l* = −1 reflection displays high‐frequency oscillations with a period similar to the oscillations at the mini‐BZ boundary discussed above. These mini‐BZ center oscillations coincide with the zone folded acoustic mode at the zone center and intensity at this frequency, thus appear across the entire duration of the measurement. The SL *l* = −1 reflection exhibits a frequency mode near *f* = 0.94 THz, slightly higher than the frequency of 0.87 THz observed at the mini‐BZ boundary at *Q*_z_ = 3.06 Å^−1^ in Figures 1e and 2c. The 0.48 THz mode is also present in the mini‐BZ‐center reflection shown in Figure 3a. Part of the experimental study was repeated at 25 °C in order to test the possibility that the accumulation of mobile charge states can lead to a perturbation of the domain configuration, as described previously. The charge carriers have more severe effects at room temperature [19, 23]. The frequency modes observed at 25 °C, however, are the same as at 120 °C (Section S5), leading us to conclude that trapping effects do not affect the oscillations at either temperature. Measurements at the SL *l* = +1 reflection exhibit contributions from acoustic oscillations in the substrate (see Section S4) and were not used to probe the non‐dispersive oscillations.

Dynamical phase‐field simulations were performed to probe the origin of the experimentally observed polarization oscillations. The simulations solved the coupled equations of motion for polarization and mechanical displacement, along with the electrostatic equilibrium equation in each time step. (PTO)*_m_*/(STO)*_n_* SLs with different *m* and *n* were simulated. The input materials parameters of the dynamical phase‐field simulations, including the mass coefficient and the damping coefficient, which are the kinetic parameters in the equation of motion for the polarization, were drawn from previous dynamical phase‐field models of polarization dynamics in (PTO)_8_/(STO)_3_ SLs [28]. A time step of 0.5 fs and a cell size of 0.4 nm were used for the simulation. Details of the dynamical phase‐field simulations are in Methods and Section S6.

**FIGURE 4:**
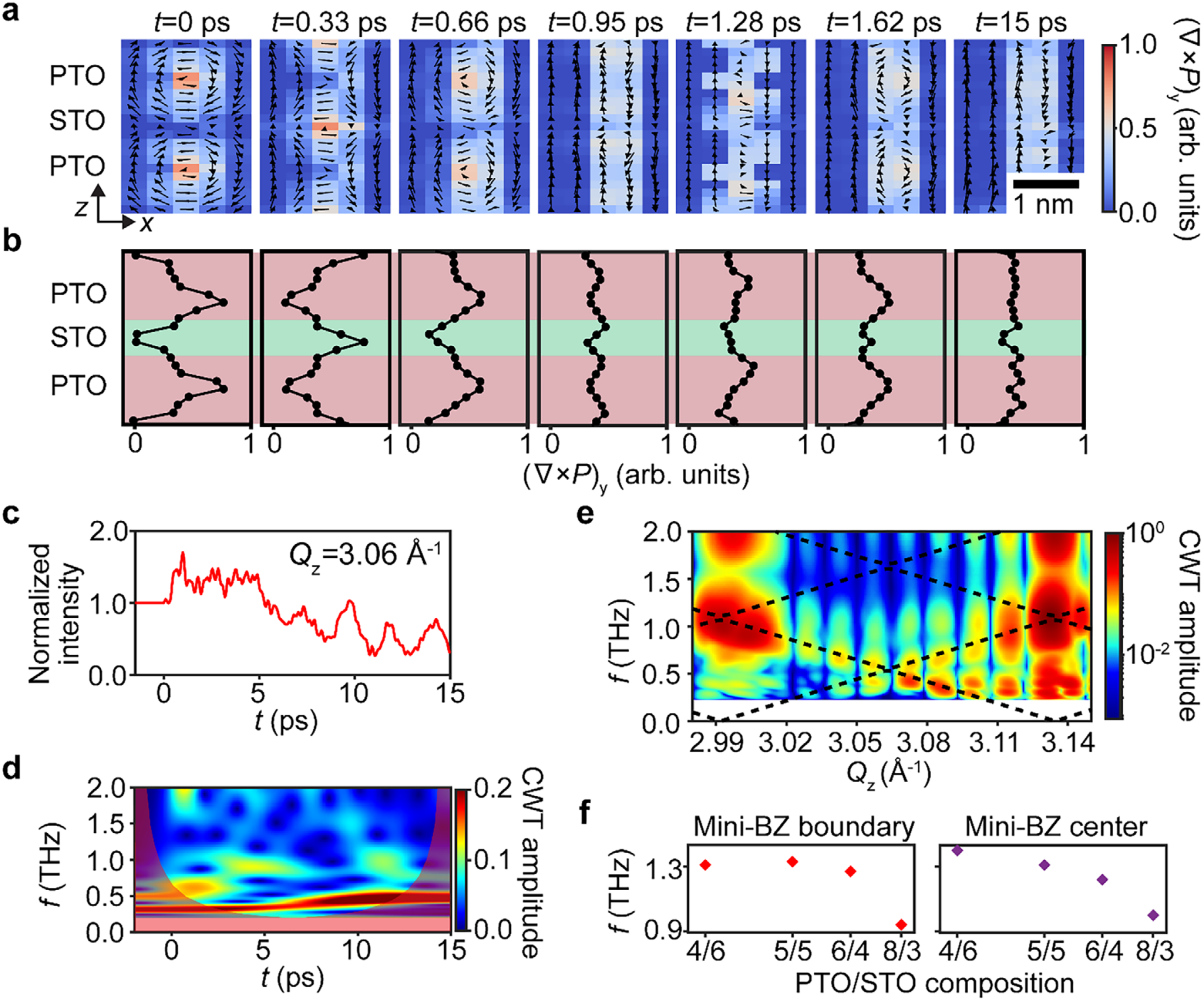
Polarization dynamics simulation and design rules. (a) Maps of simulated time‐dependence of the polarization configuration in the PTO/STO SL following the stepwise increase in the dielectric permittivity and normalized *y* component of the curl of the polarization (∇ × P)_y_. (b) (∇ × P)_y_ at the domain boundary plotted for the times shown in Figure 4a. (c) Time dependence of simulated X‐ray diffraction intensity at *Q*_z_ = 3.06 Å^−1^ calculated from the simulation results. (d) *f*‐*t* map of CWT amplitude obtained from the simulated diffracted intensity. Shaded regions correspond to areas outside the COI of the CWT. (e) Time‐averaged CWT of simulated X‐ray diffraction intensity obtained from the phase‐field simulation. (f) Frequencies of simulated (left) mini‐BZ boundary and (right) mini‐BZ center oscillations as a function of the PTO/STO composition of the SL.

The simulation results link the observed frequency modes to domain‐boundary oscillations of the ferroelectric polarization configuration. This effect is apparent in Figure 4a, which shows maps of the time dependence of the *y* component of the curl of the simulated polarization (∇ × **P**)*_y_* and of the polarization vectors within a single repeating unit of the SL. At *t* = 0, the equilibrium polarization configuration features a flux‐closure domain wall with flux‐closure‐type polarization texture centered in PTO layer. At the longest simulation time in Figure 4a, *t* = 15 ps, the polarization reaches an antiparallel configuration without vorticity, apparent from both the polarization vectors and the curl density map. The transition between the two limiting polarization configurations in Figure 4a is accompanied by polarization oscillations. The oscillation is particularly apparent near the domain boundary at the center of each polarization map in Figure 4a. The time dependence of (∇ × **P**)*_y_* along a single column of atoms near the domain boundary, Figure 4b, exhibits this oscillation.

Comparing the polarization configurations at *t* = 0 and *t* = 0.33 ps in Figure 4a reveals a high‐frequency oscillation. At *t* = 0.33 ps, the flux‐closure type configuration present at *t* = 0 within the PTO layer has largely disappeared, and the configuration of the same handedness persists in the STO layer. Consistent with this observation, the normalized curl density at *t* = 0 in Figure 4b shows a value of approximately 1 at the PTO layer center and 0 at the STO layer center. By *t* = 0.33 ps, the values of the curl density at these locations have reversed. The initial reversal of curl density in the PTO and STO layers corresponds to a short‐lived, high‐frequency (∼1.55 THz) response that completes a single oscillation cycle essentially and therefore does not appear prominently in the experimental data or in the CWT analysis.

A second, longer‐lived oscillation of the curl density follows the initial oscillation. The simulated polarization in Figure 4a evolves from a PTO‐centered flux‐closure at *t* = 0.66 ps, to antiparallel with reduced vorticity at the PTO domain boundary at *t* = 0.95 ps, and back to flux‐closure *t* = 1.62 ps in Figure 4. The similarity of the curl‐density patterns at t = 0.66 ps and t = 1.62 ps arises from this second oscillation, which has a temporal frequency of approximately 1.1 THz.

The changes in the polarization direction and magnitude of near the domain wall in Figure 4a,b occur over a range of distances in *z*. The curl density in Figure 4b shows two weak maxima near the PTO domain boundary at *t* = 0.95 ps, suggesting a possible local doubling of the effective spatial periodicity in this region. The doubling of the spatial periodicity provides a potential explanation for the higher spectral amplitude of the polarization oscillation mode near the mini‐Brillouin zone boundary. The range of spatial frequencies apparent in these transient oscillations causes their spectrum to become nearly dispersionless.

The oscillation frequency of the polarization in the dynamical phase‐field simulation was determined using an analysis similar to the experimental approach. The simulated diffracted intensity at *Q*_z_ = 3.06 Å^−1^, analogous to the experimental result in Figure 1d, is shown in Figure 4c. The computation of the diffraction patterns from the simulation is discussed in Section S7. Faster oscillations are observed at earlier times (*t*<5 ps), followed by slower oscillations over the entire time range. The CWT amplitude for the simulation, Figure 4d, reveals frequency modes at *f* = 0.4 THz, *f* = 0.5 THz, *f* = 0.66 THz, and *f* = 0.94 THz. The *f* = 0.5 THz and *f* = 0.66 THz modes correspond to the gapped acoustic phonon mode at the mini‐BZ boundary. In contrast, the *f* = 0.4 THz and *f* = 0.94 THz modes correspond to non‐equilibrium frequency modes excited by photoinduced polarization dynamics in the SL. While the 0.94 THz mode is connected to the oscillations in the phase‐field simulations, the origin of the 0.4 THz mode is less clear. The modes predicted by the dynamical phase‐field simulation have slightly different frequencies than the experimental results. We attribute this difference to the uncertainty in the mass coefficient employed in the simulation.

The wavevector dependence of the temporal oscillations in the simulation was probed by using the CWT of the time‐ and wavevector‐dependent diffraction patterns computed from the simulation results, Figure 4e. A nearly non‐dispersive mode was observed around *f* = 0.94 THz. Additionally, a mode spanning a wide range of wavevectors near *f* = 0.48 THz is also present in Figure 4e. Similar modes are observed in the experimental results in Figure 2c. Lines with velocities corresponding to the longitudinal acoustic phonon dispersion are also shown for comparison in Figure 4e.

The computational trends in the oscillation dynamics were further explored by simulating SLs with varying PTO and STO layer thicknesses while keeping the total repeating‐unit thickness approximately constant. The calculated oscillation frequencies at the mini‐BZ center and boundary are plotted as a function of layer ratio in Figure 4f for several PTO/STO layer thicknesses given in u.c.: 8/3, 6/4, 5/5, and 4/6. For the 8/3 SL discussed above, the simulations yield frequencies of 1.0 THz (at the mini‐Brillouin Zone center) and 0.94 THz (at the mini‐Brillouin Zone boundary). Reducing the PTO thickness results in a systematic increase in the calculated oscillation frequency, reaching ∼1.4 THz and ∼1.3 THz for the 4/6 SL at the mini‐Brillouin Zone center and boundary, respectively. These results reflect the expected trend that the frequency of polarization oscillation depends on the mass coefficient and the curvature of the local free‐energy landscape [29]. An identical mass coefficient is used in all of the layer combinations. The higher calculated oscillation frequency, therefore indicates that there is a higher curvature of the landscape of local free energy density in SL with lower PTO thickness. These results represent computational predictions of the tunability and illustrate a possible pathway for modifying the dynamical properties of the superlattice by varying the component layer thicknesses that can be verified in future experiments.

## 3 Conclusions

Time‐resolved X‐ray diffraction reveals that oscillatory polarization dynamics arise in a (PTO)_8_/(STO)_3_ superlattice due to depolarization field screening. The observed oscillations in the diffracted X‐ray intensity arise from modes that are not predicted by elastic models and are linked to the initial polarization state of the system. Dynamical phase‐field simulations indicate that the oscillations observed in the scattering experiment are associated with polarization reorientation at the domain walls of the PTO and STO layers, driven by the depolarization field screening by photoinduced charge carriers. The specific THz mode of oscillation observed in the simulation arises during the dynamical transition from a configuration including a flux closure feature at the domain wall to a configuration with significantly reduced closure and thus reduced vorticity. The transformation is accompanied by a lightly damped oscillation of the polarization configuration.

The optically driven polarization oscillations reported arise from depolarization effects and are thus distinct from dynamics initiated by THz electric fields. Under THz excitation, vortexon oscillations at tens to hundreds of GHz frequencies emerge in large‐scale polar vortex structures from the picosecond‐timescale reversal of vorticity, typically involving regions spanning tens of unit cells and thus manifesting as low‐frequency modes [28]. In contrast, the high‐frequency oscillations observed in the nanodomain phase originate from smaller‐scale features localized at domain boundaries, where enhanced polarization rotation leads to much higher mode frequencies. The frequency of the oscillations is in the THz regime, roughly corresponding to elastic phenomena at the size scale of the SL period, and at far lower frequencies than the soft mode associated with the unit‐cell scale development of the ferroelectric polarization.

An additional critical difference between THz excitation and optical excitation is that under optical excitation, the polarization is dramatically altered, and it no longer serves as a conserved quantity of the topological configuration. The topological conservation thus becomes far less important and allows the rapid disappearance of features such as the closure polarization feature and, potentially, vorticity.

The dynamical polarization behavior in this model system within the broader range of FE/DE SLs reveals that optical impulses can excite oscillations of long‐range and topological polarization order (e.g., the near‐domain wall polarization in the present case). The oscillations can be systematically varied using the principles of FE/DE SL design, including relative thicknesses of PTO and STO. With this insight, it may be possible to design THz polarization oscillations with specific frequencies that could be selected, for example, to resonate with magnetic or structural modes. Further detailed study of the oscillations will reveal design principles for tuning the THz oscillation frequency of FE/DE SLs and could further provide the means to stabilize the crucial intermediate polarization configuration, for example, using applied electric fields. The further understanding of non‐equilibrium phonon dynamics and polarization phenomena in ferroelectric SLs and thin films can include the effects of varying polarization screening phenomena and could result in further novel coupled modes of excitation. The results also provide the means to connect frequency‐resolved or spectroscopic measurements to the nanoscopic polarization configuration.

## 4 Methods

### 4.1 Time‐Resolved X‐Ray Diffraction Measurement

The experiments reported here probed a SL consisting of 23 repeats of alternating layers of 8 u. c. of the FE oxide PTO, and 3 u. c. of the DE oxide STO, an overall thickness of 100 nm, grown on an SRO bottom electrode on an (001)‐oriented STO substrate [30]. The structural perturbations induced by the optical pump were studied at the X‐ray Pump‐Probe beamline of Linac Coherent Light Source (LCLS) [31]. The SL was excited using a *π*‐polarized optical pulse with a wavelength of 400 nm. The optical photon energy was slightly below the nominal absorption edge of PTO and STO, and the excitation was thus uniformly distributed throughout the thickness of the SL [32, 33, 34]. The diffraction patterns were recorded using a 9.5 keV X‐ray probe pulse with a repetition rate of 120 Hz with a pulse duration of 40 fs. The footprints of the optical pump and x‐ray probe on the sample were 240 × 620 µm^2^ and 30 × 80 µm^2^, respectively. Previous x‐ray nanobeam diffraction studies have shown that the total intensity, domain period, and domain coherence length are constant on the sub‐micron to micron scale [35]. We thus do not expect significant variation across the footprint of the X‐ray beam in this measurement. Frequencies are reported in terms of the temporal frequency *f*, the inverse of one oscillation period. In addition to optical absorption directly in the SL, resulting in the effects described here, absorption in the metallic SRO bottom electrode also results in an increase in the temperature of the SRO layer and produces an acoustic pulse arising from thermal expansion that produces oscillations that persist for the entire acoustic round‐trip time of 50 ps [21]. The steady‐state diffraction pattern in Figure 2a was acquired at station 7‐ID‐C of the Advanced Photon Source at Argonne National Laboratory.

### 4.2 Dynamical Phase‐Field Simulations

The dynamical phase‐field simulations described the PTO/STO SL with a simulation volume with total size 120Δ*x* × 120Δ*y* × *N*_z_Δz using a cell size with Δ*x =*Δ*y =*Δ*z* = 0.4 nm. Along the out‐of‐plane direction (*z* axis), from bottom to top, the thicknesses of the STO substrate, SrRuO_3_ (SRO) electrode, the [(PTO)*_m_*/(STO)*_n_*]*_p_* superlattice, and air layer were 50, 10, *p*(*m*+*n*), and 20 cells, respectively, with *p* = 10. The background dielectric permittivity κ_b_ of the STO layer was rapidly changed from 40 to 10^4^ at *t* = 0 ps, and the coupled equations of motion for polarization and mechanical displacement were solved. These equations were solved using the central finite difference method for spatial derivatives, with a midpoint derivative approximation, and the classical Runge‐Kutta method for time integration, with a time step of Δ*t* = 0.5 fs. Halving Δ*t* did not alter the polarization and strain dynamics. The specific forms and solution procedures of the governing equations, the detailed free energy expansions, materials parameters, and the implementation of boundary conditions are described in the Section S6.

## Author Contributions

D.S.G, H.J.L., M.D., and P.G.E. conceived the experiment. X‐ray diffraction measurements were conducted by D.S.G., H.J.L., Y.A., S.D.M., J.M.G., D.Z., T.S., S.S., H.W., and P.G.E. Ferroelectric/dielectric superlattice specimens were synthesized by M.H.Y. and M.D. X.G. performed the dynamical phase‐field simulations under the supervision of J.‐M.H. All authors participated in the analysis, interpretation, and discussion of the results. The initial draft of the manuscript was written by D.S.G. and subsequently revised by all authors except M.H.Y.

## Conflicts of Interest

The authors declare no conflicts of interest.

## Supporting information




**Supporting File**: advs73872‐sup‐0001‐SuppMat.docx.

## Data Availability

The data and computational results presented in this article are available by request to the corresponding author.

## References

[1] T. Kampfrath , K. Tanaka , and K. A. Nelson , “Resonant and Nonresonant Control Over Matter and Light by Intense Terahertz Transients,” Nature Photonics 7 (2013): 680–690, 10.1038/nphoton.2013.184.

[2] T. Nagatsuma , G. Ducournau , and C. C. Renaud , “Advances in Terahertz Communications Accelerated by Photonics,” Nature Photonics 10 (2016): 371–379, 10.1038/nphoton.2016.65.

[3] H. T. Chen , W. J. Padilla , J. M. O. Zide , A. C. Gossard , A. J. Taylor , and R. D. Averitt , “Active Terahertz Metamaterial Devices,” Nature 444 (2006): 597–600, 10.1038/nature05343. 17136089

[4] H. Tao , C. M. Bingham , and A. C. Strikwerda , et al., “Highly Flexible Wide Angle of Incidence Terahertz Metamaterial Absorber: Design, Fabrication, and Characterization,” Physical Review B 78 (2008): 241103, 10.1103/PhysRevB.78.241103.

[5] J. Xue , C. Chen , and S. Tian , et al., “Control and Optimization of Absorbing Behavior in Graphene‐Based Multiple Narrowband Metamaterial Absorber by Machine Learning,” Optics Communications 587 (2025): 131958, 10.1016/j.optcom.2025.131958.

[6] J. Zou , E. Thingstad , J. S. Seo , S. K. Kim , J. Klinovaja , and D. Loss , “Tunable Ultrafast Dynamics of Antiferromagnetic Vortices in Nanoscale Dots,” Physical Review Research 7 (2025): 023023, 10.1103/PhysRevResearch.7.023023.

[7] D. Kaplan , P. A. Volkov , A. Chakraborty , Z. Zhuang , and P. Chandra , “Tunable Spatiotemporal Orders in Driven Insulators,” Physical Review Letters 134 (2025): 066902, 10.1103/PhysRevLett.134.066902. 40021154

[8] S. Zheng , C. Liu , Y. Zhang , et al., “Giant Shear‐Vertical Wave Bandgaps Induced by Diffuse Domain‐Walls in Ferroelectrics,” International Journal of Mechanical Sciences 294 (2025): 110239, 10.1016/j.ijmecsci.2025.110239.

[9] L. Zhang , Q. Zhao , A. Jain , and T. Koschny , “Laser Heating of Ferroelectric Cubes for Tunable Electromagnetic Properties in Metamaterials,” Applied Physics Letters 123 (2023): 211703, 10.1063/5.0176424.

[10] S. Das , Y. Tang , Z. Hong , et al., “Observation of Room‐Temperature Polar Skyrmions,” Nature 568 (2019): 368–372, 10.1038/s41586-019-1092-8. 30996320

[11] P. Shafer , P. García‐Fernández , P. Aguado‐Puente , et al., “Emergent Chirality in the Electric Polarization Texture of Titanate Superlattices,” Proceedings of the National Academy of Sciences 115 (2018): 915–920, 10.1073/pnas.1711652115. PMC5798329 29339493

[12] A. Yadav , C. Nelson , S. Hsu , et al., “Observation of Polar Vortices in Oxide Superlattices,” Nature 530 (2016): 198–201, 10.1038/nature16463. 26814971

[13] M. Dawber , C. Lichtensteiger , M. Cantoni , et al., “Unusual Behavior of the Ferroelectric Polarization in PbTiO_3_/SrTiO_3_ Superlattices,” Physical Review Letters 95 (2005): 177601, 10.1103/PhysRevLett.95.177601. 16383870

[14] M. Dawber and E. Bousquet , “New developments in Artificially Layered Ferroelectric Oxide Superlattices,” MRS Bulletin 38 (2013): 1048–1055, 10.1557/mrs.2013.263.

[15] J. Gregg , “Exotic Domain States in Ferroelectrics: Searching for Vortices and Skyrmions,” Ferroelectrics 433 (2012): 74–87, 10.1080/00150193.2012.678131.

[16] H. H. Wang , V. A. Stoica , C. Dai , et al., “Terahertz‐Field Activation of Polar Skyrons,” Nature Communications 16 (2025): 8994, 10.1038/s41467-025-64033-6. PMC12511383 41068083

[17] W. Li , S. Wang , P. Peng , et al., “Terahertz Excitation of Collective Dynamics of Polar Skyrmions Over A Broad Temperature Range,” Nature Physics 21 (2025): 1965–1972, 10.1038/s41567-025-03056-8.

[18] R. Gu , R. Xu , F. Delodovici et al., “Superorders and Terahertz Acoustic Modes in Multiferroic BiFeO_3_/LaFeO_3_ Superlattices” Applied Physics Reviews 2024, 11, 041415.

[19] Y. Ahn , J. Park , A. Pateras , et al., “Photoinduced Domain Pattern Transformation in Ferroelectric‐Dielectric Superlattices,” Physical Review Letters 119 (2017): 057601, 10.1103/PhysRevLett.119.057601. 28949700

[20] B. Guzelturk , T. Yang , Y.‐C. Liu , et al., “Sub‐Nanosecond Reconfiguration of Ferroelectric Domains in Bismuth Ferrite,” Advanced Materials 35 (2023): 2306029, 10.1002/adma.202306029. 37611614

[21] H. J. Lee , Y. Ahn , S. D. Marks , et al., “Structural Evidence for Ultrafast Polarization Rotation in Ferroelectric/Dielectric Superlattice Nanodomains,” Physical Review X 11 (2021): 031031.

[22] J. Y. Jo , P. Chen , R. J. Sichel , et al., “Nanosecond Dynamics of Ferroelectric/Dielectric Superlattices,” Physical Review Letters 107 (2011): 055501, 10.1103/PhysRevLett.107.055501. 21867078

[23] J. Park , Y. Ahn , J. A. Tilka , et al., “Role of temperature‐Dependent Electron Trapping Dynamics in the Optically Driven Nanodomain Transformation in A PbTiO_3_/SrTiO_3_ Superlattice,” Applied Physics Letters 116 (2020): 012901, 10.1063/1.5128364.

[24] D. Reis , M. DeCamp , P. Bucksbaum , et al., “Probing Impulsive Strain Propagation With X‐Ray Pulses,” Physical Review Letters 86 (2001): 3072, 10.1103/PhysRevLett.86.3072. 11290110

[25] J. M. Lilly , “Element Analysis: A Wavelet‐Based Method for Analysing Time‐Localized Events in Noisy Time Series,” Proceedings of the Royal Society A: Mathematical, Physical and Engineering Sciences 473 (2017): 20160776, 10.1098/rspa.2016.0776. PMC5415685 28484325

[26] T. Vasileiadis , J. Varghese , V. Babacic , J. Gomis‐Bresco , D. Navarro Urrios , and B. Graczykowski , “Progress and Perspectives on Phononic Crystals,” Journal of Applied Physics 129 (2021): 160901, 10.1063/5.0042337.

[27] C. Colvard , T. Gant , M. Klein , et al., “Folded Acoustic and Quantized Optic Phonons in (GaAl)As Superlattices,” Physical Review B 31 (1985): 2080, 10.1103/PhysRevB.31.2080. 9936014

[28] Q. Li , V. A. Stoica , M. Paściak , et al., “Subterahertz Collective Dynamics of Polar Vortices,” Nature 592 (2021): 376–380, 10.1038/s41586-021-03342-4. 33854251

[29] Y. Zhu , T. Chen , A. Ross , et al., “Theory of Nonlinear Terahertz Susceptibility in Ferroelectrics,” Physical Review B 110 (2024): 054311, 10.1103/PhysRevB.110.054311.

[30] S. K. Streiffer , J. A. Eastman , D. D. Fong , et al., “Observation of Nanoscale 180° Stripe Domains in Ferroelectric PbTiO_3_ Thin Films,” Physical Review Letters 89 (2002): 067601, 10.1103/PhysRevLett.89.067601. 12190610

[31] M. Chollet , R. Alonso‐Mori , M. Cammarata , et al., “The X‐ray Pump–Probe Instrument at the Linac Coherent Light Source,” Journal of Synchrotron Radiation 22 (2015): 503–507, 10.1107/S1600577515005135. 25931060 PMC4416667

[32] A. J. A. El‐Haija , “Effective Medium Approximation for the Effective Optical Constants of a Bilayer and A Multilayer Structure Based on the Characteristic Matrix Technique,” Journal of Applied Physics 93 (2003): 2590–2594, 10.1063/1.1543229.

[33] M. Cardona , “Optical Properties and Band Structure of SrTiO_3_ and BaTiO_3_ ,” Physical Review 140 (1965): A651, 10.1103/PhysRev.140.A651. 10004703

[34] N. Izyumskaya , V. Avrutin , X. Gu , et al., “Structural and Optical Properties of PbTiO_3_ Grown on SrTiO_3_ Substrates by Peroxide MBE,” in Materials Research Society (MRS) Online Proceedings Library 966 (Cambridge University Press 2007): 704, 10.1557/PROC-0966-T07-04.

[35] J. Park , J. Mangeri , Q. T. Zhang , et al., “Domain Alignment Within Ferroelectric/Dielectric PbTiO_3_ /SrTiO_3_ Superlattice Nanostructures,” Nanoscale 10 (2018): 3262–3271, 10.1039/C7NR07203A. 29384166

[36] J. M. Lilly and S. C. Olhede , “Generalized Morse Wavelets as A Superfamily of Analytic Wavelets,” IEEE Transactions on Signal Processing 60 (2012): 6036–6041, 10.1109/TSP.2012.2210890.

[37] T. Yang , B. Wang , J.‐M. Hu , and L.‐Q. Chen , “Domain Dynamics Under Ultrafast Electric‐Field Pulses,” Physical Review Letters 124 (2020): 107601, 10.1103/PhysRevLett.124.107601. 32216398

[38] B. Cheng , P. L. Kramer , Z.‐X. Shen , and M. C. Hoffmann , “Terahertz‐Driven Local Dipolar Correlation in a Quantum Paraelectric,” Physical Review Letters 130 (2023): 126902, 10.1103/PhysRevLett.130.126902. 37027861

[39] P. Tang , R. Iguchi , K.‐I. Uchida , and G. E. W. Bauer , “Excitations of the Ferroelectric Order,” Physical Review B 106 (2022): L081105.

[40] Y. Li , S. Hu , Z. Liu , and L. Chen , “Effect of substrate constraint on the Stability and Evolution of Ferroelectric Domain Structures in Thin Films,” Acta Materialia 50 (2002): 395, 10.1016/S1359-6454(01)00360-3.

[41] L.‐Q. Chen , "Landau Free‐Energy Coefficients," in Physics of Ferroelectrics: A Modern Perspective, eds. K. M. Rabe , C. H. Ahn , and J.‐M. Triscone (Springer, 2007): 363–372.

[42] A. P. Levanyuk , B. A. Strukov , and A. Cano , “Background Dielectric Permittivity: Material Constant or Fitting Parameter?,” Ferroelectrics 503 (2016): 94–103, 10.1080/00150193.2016.1218245.

[43] D. C. Ma , Y. Zheng , and C. H. Woo , “Phase‐Field Simulation of Domain Structure for PbTiO_3_/SrTiO_3_ Superlattices,” Acta Materialia 57 (2009): 4736–4744, 10.1016/j.actamat.2009.06.032.

[44] J. Wang , X. Ma , Q. Li , J. Britson , and L.‐Q. Chen , “Phase Transitions and Domain Structures of Ferroelectric Nanoparticles: Phase Field Model Incorporating Strong Elastic and Dielectric Inhomogeneity,” Acta Materialia 61 (2013): 7591–7603, 10.1016/j.actamat.2013.08.055.

[45] T. Hasegawa , S.‐I. Mouri , Y. Yamada , and K. Tanaka , “Giant Photo‐Induced Dielectricity in SrTiO_3_ ,” Journal of the Physical Society of Japan 72 (2003): 41–44, 10.1143/JPSJ.72.41.

[46] V. Stoica , N. Laanait , C. Dai , et al., “Optical Creation of a Supercrystal With Three‐Dimensional Nanoscale Periodicity,” Nature Materials 18 (2019): 377–383, 10.1038/s41563-019-0311-x. 30886403

[47] D. Gerlich and E. Fisher , “The High Temperature Elastic Moduli of Aluminum,” Journal of Physics and Chemistry of Solids 30 (1969): 1197–1205, 10.1016/0022-3697(69)90377-1.

[48] G. Rupprecht and R. Bell , “Dielectric Constant in Paraelectric Perovskites,” Physical Review 135 (1964): A748, 10.1103/PhysRev.135.A748.

[49] T. Chen , B. Wang , Y. Zhu , S. Zhuang , L.‐Q. Chen , and J.‐M. Hu , “Analytical Model and Dynamical Phase‐Field Simulation of Terahertz Transmission Across Ferroelectrics,” Physical Review B 109 (2024): 094305, 10.1103/PhysRevB.109.094305.

[50] P. Aguado‐Puente and J. Junquera , “Structural and Energetic Properties of Domains in PbTiO_3_/SrTiO_3_ Superlattices From First Principles,” Physical Review B 85 (2012): 184105, 10.1103/PhysRevB.85.184105.

[51] E. Zatterin , P. Ondrejkovic , L. Bastogne , et al., “Assessing the Ubiquity of Bloch Domain Walls in Ferroelectric Lead Titanate Superlattices,” Physical Review X 14 (2024): 041052.

