# Supplementary material for: Transient Terahertz Oscillations During Photoinduced Polarization Topology Reconfiguration in Ferroelectric Superlattices
Source: Adv Sci (Weinh). 2026 Feb 3;13(30):e22387. doi: 10.1002/advs.202522387 (PMC13248806; doi:10.1002/advs.202522387)
Supplement: Supplementary file 1 — Supporting File: advs73872‐sup‐0001‐SuppMat.docx. [file ADVS-13-e22387-s001.docx]

**Supporting Information for Transient Terahertz Oscillations During Photoinduced Polarization Topology Reconfiguration in Ferroelectric Superlattices**

*Deepankar Sri Gyan^1^, Hyeon Jun Lee^1,2^,* *Xiangwei Guo^1^, Youngjun Ahn^1,3^, Samuel D. Marks^1^, Mohammed H. Yusuf^4^, Matthew Dawber^4,5^, James M. Glownia^6^, Diling Zhu^6^, Takahiro Sato^6^, Sanghoon Song^6^_,_ Haidan Wen^3,7^, Jia-Mian Hu^1^, and Paul G. Evans^1*^*

*^1^ Department of Materials Science and Engineering, University of Wisconsin-Madison, Madison, Wisconsin 53706, USA*

*^2^* *Department of Materials Science and Engineering, Kangwon National University, Samcheok, 25913, South Korea*

*^3^ Materials Science Division*, *Argonne National Laboratory, Lemont, Illinois 60439, USA*

*^4^ Department of Physics and Astronomy, Stony Brook University, Stony Brook, New York 11794, USA*

*^5^ Department of Physics, University of Vermont, Burlington, Vermont 05405. USA*

*^6^ Linac Coherent Light Source, SLAC National Accelerator Laboratory, Menlo Park, California 94025, USA*

*^7^ X-ray Science Division*, *Argonne National Laboratory, Lemont, Illinois 60439, USA*

** Electronic mail: pgevans@wisc.edu*

***Calculation of longitudinal acoustic phonon dispersion***

**Figure S1** shows a diagram of a single repeating unit of the SL. The strain distribution within this repeating unit is broken into several components to facilitate the numerical calculation of the strain pulse. The PTO/STO repeating units are labeled with index *i*, where *i* has values 1 and the total number of repeating units. The strain propagating into repeating unit *i* from the PTO layer beneath it, i.e. the PTO layer of repeating unit *i*-1 is defined to be *ε*_in, PTO_. Upon reaching the PTO/STO interface the upward propagating strain is divided into components 1) propagating into the STO layer (*ε*_in, STO_), and 2) reflected from the lower PTO/STO interface (*ε*_ref, STO_). The *ε*_in, STO_ component again splits into two components upon reaching the upper STO/PTO interface: *ε*_in, PTO,_ and *ε*_ref, PTO_.


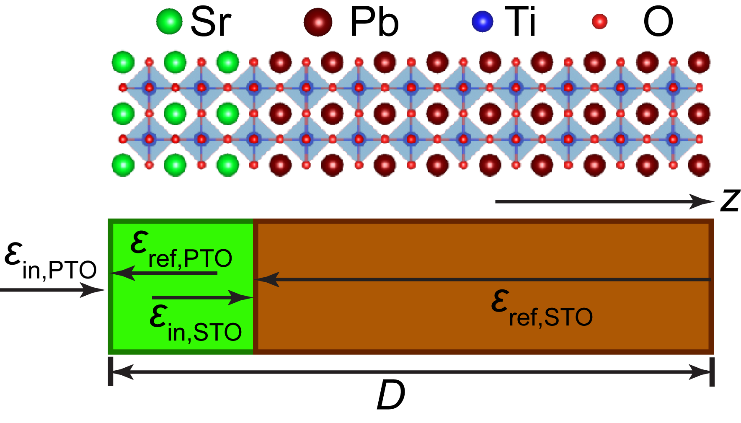


**Figure S1**. **Elastic model of SL lattice dynamics.** (top) Schematic atomic structure of a single repeating unit of the 8-PbTiO_3_/3-SrTiO_3_ SL. (bottom) Definitions of components of the strain employed in the analysis of the acoustic pulse.

The interference between the upward and downward propagating strain pulses results in a complex distribution of vibrational modes which can be predicted using a continuum mechanical model. The acoustic pulses are described by the displacement field *u*(*z*,*t*). The displacement field is related to the stress field *σ*(*z,t*) by:^[27]^

$\rho\left( z \right)\frac{\partial^{2}u\left( z,t \right)}{\partial t^{2}}=\frac{\partial\sigma(z,t)}{\partial z}$ (S1)

Here *z* is the depth with respect to the free surface, *ρ*(*z*) is the density at depth *z*, and *t* is time. Solving **equation S1** gives a relationship between the wavevector *q*_z_ and angular frequency *ω*:^[27]^

$\cos\left( q_{z} D \right)=\cos\left( \frac{\omega d_{PTO}}{v_{PTO}} \right)\cos\left( \frac{\omega d_{STO}}{v_{STO}} \right)-\frac{1}{2}(Z_{PTO/STO}+\frac{1}{Z_{PTO/STO}} ) sin\left( \frac{\omega d_{PTO}}{v_{PTO}} \right)\sin\left( \frac{\omega d_{STO}}{v_{STO}} \right)$ (S2)

Here *D* = *d*_A_+*d*_B_ is the repeating layer thickness, and *d*_A_ and *v*_A_ are the thickness and longitudinal sound velocities of layer *A,* respectively, with similar definitions for layer B. The acoustic impedance mismatch between the PTO and STO layers is *Z*_PTO/STO_ = (*ρ*_PTO_*v*_PTO_)/(*ρ*_STO_*v*_STO_). The parameters used for the calculation are in **Table S1**. The calculated phonon dispersion is shown in Figure 1b.

| **Parameters** | **Value** |
| --- | --- |
| ***d*_PTO_** | **2.96 nm** |
| ***d*_STO_** | **1.11 nm** |
| ***v*_PTO_** | **4200 m s^-1^** |
| ***v*_STO_** | **7800 m s^-1^** |
| ***ρ*_PTO_** | **7.52 g cm^-3^** |
| ***ρ*_STO_** | - 1. **g cm^-3^** |

**Table S1. Parameters for calculation of acoustic phonon dispersion and sound velocity.**

The longitudinal acoustic sound velocity in the elastic continuum model is:

$v_{PTO/STO, predicted}={D\left[ \frac{d_{PTO}^{2}}{v_{STO}^{2}}+\frac{d_{STO}^{2}}{v_{STO}^{2}}+\left( Z_{PTO/STO}+\frac{1}{Z_{PTO/STO}} \right)\frac{d_{PTO}d_{STO}}{v_{PTO}v_{STO}} \right]}^{-\frac{1}{2}}$ (S3)

The value of *v*_PTO/STO,predicted_ for the PTO/STO SL using **equation S3** and the materials parameters in Table S1 is 4700 m s^-1^. The measured sound velocity from the data shown in Figure 1e is 3800 m s^-1^. This velocity is also apparent in the longitudinal acoustic dispersion found using the time-averaged continuous wavelet transform (CWT) of the scattered X-ray intensity around the SL *l* = -1 reflection shown in **Figure S2**. The line in Figure S2 corresponds to the longitudinal acoustic phonon dispersion with a velocity of 3800 m s^-1^.


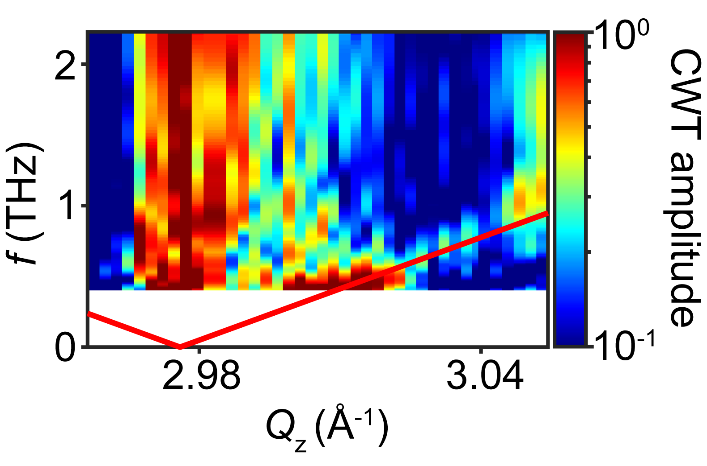


**Figure S2**. **Acoustic dispersion and dynamics near SL *l* = -1 reflection.** CWT map near SL *l* = -1 reflection taken from the *l* = -1 region of Figure 2c. The red line corresponds to the longitudinal acoustic dispersion for v_PTO/STO_ = 3800 m s^-1^.

The velocity predicted by the continuum elastic model, 4700 m s^-1^, is significantly different from the experimentally measured value, 3800 m s^-1^. The difference could arise because the sound velocity calculated using the continuum elastic model in equation S3 does not consider differences in bonding at the PTO/STO interface.

***Continuous wavelet transform analysis***

The CWT analysis was performed using a Morse wavelet in the MATLAB R2022a software package (Mathworks, Inc.).^[25, 36]^ The minimum and maximum frequencies are calculated by that package, considering the measured time range and the time resolution of the measurement. The transform analysis includes edge artifacts as a result of the limited time range. The CWT analysis also predicts a cone of influence (COI), indicating the time-dependent range of frequencies for which the CWT analysis is reliable.^[25]^ The amplitude in the frequency-time region outside the COI is less reliable.

The minimum and maximum frequencies for the CWT analysis performed on the data shown in Figure 1e were 4.34 THz and 0.14 THz. The analysis performed on the intensity map shown in Figure 2 has minimum and maximum frequencies of 0.22 and 2.18 THz. The frequency resolution was chosen by selecting the number of points per octave to be 48.

***Frequency gap at mini-BZ boundary with acoustic impedance mismatch***


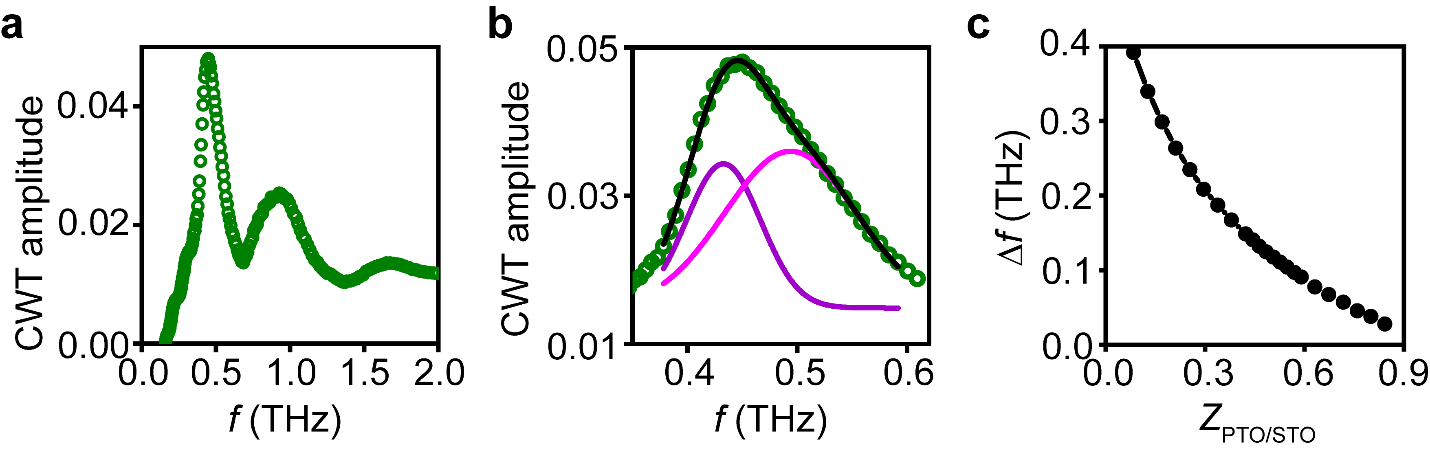


**Figure S3. Mini-BZ-boundary modes of structural dynamics.** a) Time-averaged CWT amplitude as a function of frequency computed from Figure 1e. b) CWT amplitude in a narrow frequency range near mini-BZ boundary in the time*-*averaged CWT amplitude (points). The fit (black line) is the sum of two individual gaussians (purple and magenta lines). c) Frequency gap (Δ*f*) at *Q*_z_ = 3.06 Å­­­­^‑^1 predicted using **equation S2** as a function of acoustic impedance mismatch at PTO/STO interface (*Z*_PTO/STO_).

The CWT analysis of the intensity data from Figure 1e also provides insight into the dispersion of the zone-folded acoustic modes near the center and boundaries of the mini-BZ. The time-averaged CWT amplitude map shown in Figure 1e is shown in **Figure S3**a. In addition to the zone boundary mode observed at *f* = 0.87 THz, two additional amplitude maxima are evident at *f* = 0.43 THz and 1.67 THz. These two maxima have frequency values that are close to the predicted frequencies in the region near the BZ boundary, in which the dispersion is expected to have a gap. The frequency magnitude of the gap was determined by fitting the amplitude maximum at 0.43 THz using a sum of two Gaussians, each defined as:

$$CWT amplitude=y_{0}+A\exp\left( -0.5\left( \frac{f-f_{c}}{w} \right)^{2} \right)$$

Here *y*_0_ is the background, *A* is the amplitude, *f*_c_ is the peak center, and *w* is the full width at half maximum of the peak. The results of the fit are plotted in Figure S3b. The fitted peak parameters for the two peaks are given in **Table S2**. The frequencies obtained from the fit were 0.43 THz and 0.49 THz.

The frequency gap (Δ*f*) as a function of acoustic impedance mismatch at the PTO/STO interface (*Z*_PTO/STO_) is plotted in Figure S3c. The gap predicted using the elastic continuum model for *Q*_z_ = 3.06 Å^-1^ is Δ*f* = 0.03 THz. The gap observed in the experimental data is Δ*f* = 0.06 THz. The value of *Z*_PTO/STO_ that would correspond to the observed value of Δ*f*, 0.06 THz, is 0.7, which is 20% lower than the value of 0.84 predicted using the elastic parameters of PTO and STO.

| **Parameter** | **Peak 1** | **Peak 2** |
| --- | --- | --- |
| ***y*_0_** | **0.015** | **0.015** |
| ***H*** | **0.019** | **0.021** |
| ***f*_c_ (THz)** | **0.43** | **0.49** |
| ***w*(THz)** | **0.03** | **0.06** |

**Table S2.** Parameters extracted from fitting the peak in Figure S4b using the sum of gaussians.

***Analysis of acoustic oscillations***


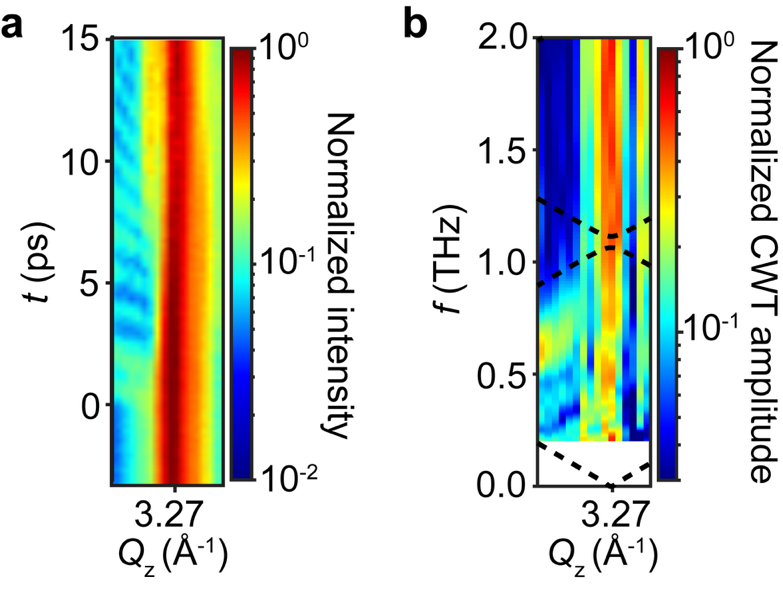


**Figure S4. Dynamics near SL *l* = +1 reflection.** a) Diffracted X-ray intensity as a function of *Q_z_* and *t* near the mini-BZ centered at the SL *l* = +1 reflection at 3.27 Å^-1^. b) Time-averaged CWT of time and wavevector-dependent intensity map around SL *l* = +1 reflection. The predicted acoustic dispersion of the SL is shown as dashed line. The substrate longitudinal acoustic oscillations from the substrate appear as amplitude between 0.5 and 1 THz at *Q_z_* < 3.27 Å^-1^.

The complete mini-BZ region between the SL Bragg (*l* = 0) and SL *l* = +1 reflection was not measured in the experiments because this range of wavevectors includes an intense contribution to the scattering from the 002 reflection of the STO substrate. Maps of diffracted X-ray intensity as a function of wavevector *Q*_z_ and time *t* in the range between -3 and 15 ps centered at SL *l* = +1 reflection is shown in **Figure S4**a. Notably, intensities around the SL *l* = +1 reflections display strong oscillations for *Q*_z_ less than 3.27 Å^-1^. The result of CWT analysis of the time-dependent intensity at the values of *Q*_z_ shown in Figure S4a is shown in Figure S4b. In addition to the principle acoustic SL mode, a dominant contribution was observed in the form of a dispersive phonon branch originating from the longitudinal acoustic phonon modes of the STO substrate.

***Polarization oscillations at 25 ºC.***


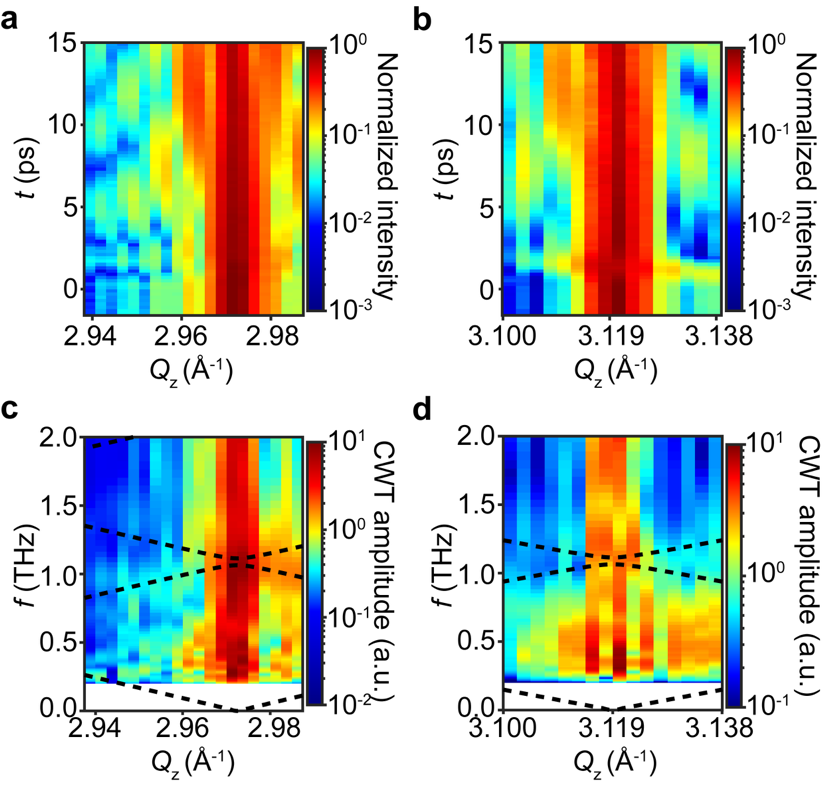


**Figure S5. Dynamics at 25 ºC.** Diffracted X-ray intensity as a function of *Q*_z_ and *t* at room temperature near the a) SL *l* = -1 and b) SL *l* = 0 reflections. Time-averaged CWT amplitude as a function of *Q*_z_ and *f* for the c) SL *l* = -1 and d) SL *l* = 0 reflections.

The X-ray intensity distribution near SL *l* = -1 and SL *l* = 0 reflections measured at 25 ºC are shown in **Figure S5**a and S5b, respectively. The measurements show that there are slower acoustic oscillations for the entire *t* along with faster oscillations for *t*<2 ps. A CWT analysis for the intensity in this region is shown in Figure S5c and S5d. Oscillations with frequencies near 0.95 THz are apparent in Figure S5c and are consistent with the oscillations observed at 120 ℃. The 0.48 THz frequency mode observed at 120 ℃ is also present at room temperature.

The top panels of **Figure S6**a and, S6b show the intensities measured at 120 ℃ at individual wavevectors corresponding to the *l* = 0 and +1 reflections, respectively. Similar measurements conducted at room temperature are shown in the top panel of Figure S6c and S6d, respectively. The X-ray intensity in each panel of Figure S6 is normalized to the value before *t* = 0*.* The bottom panels of Figure S6 shows CWT maps computed from the intensities in the top panels. The SL *l* = 0 reflection, Figure S6a and S6c, has a single oscillation period immediately following optical excitation. Additional high-frequency oscillations for *t* > 5 ps, arise from the propagation of the photoacoustic strain pulse in the substrate. The intensity of the SL *l* = +1 reflection, Figure S6b and S6d have high-frequency oscillations with a period similar to those of the *l* = -1 reflection shown in Figure 3 of the text. The *f*-*t* maps for the SL *l* = +1 reflection, Figure S6b and S6d prominently display the sharp 0.6 THz frequency mode due to contributions from the STO substrate at that specific *Q*_z_.


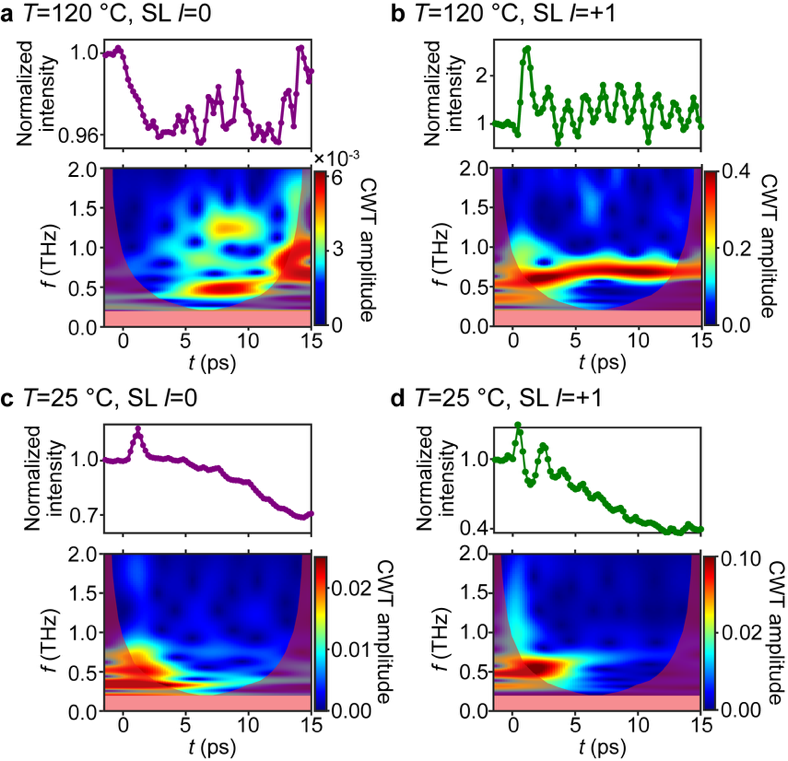


**Figure S6. Dynamics of SL *l* = 0 reflection and appearance of substrate acoustic oscillations near SL *l* = +1 reflection.** Intensity as a function of time and the CWT *f* - *t* maps for the a) SL *l* = 0 reflection at *T* = 120 ℃, b) SL *l* = +1 reflection at *T* = 120 ℃, c) SL *l* = 0 reflection at *T* = 25 ℃, and d) SL *l* = +1 reflection at *T* = 25 ℃.

***Dynamical phase-field simulations***

The dynamical evolution of the lattice polarization *P_i_* (*i* = *x*, *y*, *z*) in the (001)_pc_ PTO/STO SL (pc: pseudocubic) grown on the SRO-coated (001)-oriented STO substrate is governed by:^[28-29, 37]^

$\mu\frac{\partial^{2}P_{i}}{\partial t^{2}}+\gamma\frac{\partial P_{i}}{\partial t}=-\frac{\delta F}{\delta P_{i}}$, (S4)

with *i*=*x*, *y*, or *z*. Here, *μ* is the mass coefficient that is related to the reduced mass and Born effective charge of the soft mode as well as the volume of the unit cell.^[38-39]^ *γ* is the phenomenological damping coefficient, respectively, with *x*||[100]_c_, *y*||[010]_c_, and *z*||[001]_c_ (c: cubic). The electric Helmholtz free energy *F* of the PTO/STO system is expressed as:

$F\left( T,P_{i},E_{i},\varepsilon_{ij} \right) =\int(g_{0}\left( T \right)+f_{\mathrm{Landau}}(P_{i})+f_{\mathrm{grad}}(P_{i,j})+f_{\mathrm{elec}}({P_{i},E}_{i})+f_{\mathrm{elas}}(P_{i},\varepsilon_{ij}))dV$, (S5)

where $g_{0}\left( T \right)$ is the Gibbs free energy density of the non-polar reference phase with zero spontaneous polarization, zero electric field, and zero stress. The Landau free energy density $f_{\mathrm{Landau}}$ describes the change in the Gibbs free energy density due to the formation of a lattice polarization *P_i_* in the absence of electric and stress fields, and is written as:

$f_{\mathrm{Landau}}=\alpha_{ij}P_{i}P_{j}+\alpha_{ijkl}P_{i}P_{j}P_{k}P_{l}+\alpha_{ijklmn}P_{i}P_{j}P_{k}P_{l}P_{m}P_{n}$, (S6)

where the $\alpha_{ij}$, $\alpha_{ijkl}$ and $\alpha_{ijklmn}$ are the Landau coefficients under stress-free conditions. The expressions of $f_{\mathrm{Landau}}$ for PTO and STO can be found in refs. ^[40-41]^.

The second term is the gradient energy density:

$f_{\mathrm{grad}}=\frac{1}{2}G_{11}\left( \nabla\mathbf{P} \right)^{2}$, (S7)

where $G_{11}$ is the isotropic gradient coefficient.

The third term is the electrostatic energy density,

$f_{\mathrm{elec}}=-\frac{1}{2}\kappa_{0}\kappa_{b}E_{i}E_{j}-E_{i}P_{i}$, (S8)

where $\kappa_{0}$ is the vacuum permittivity, and $\kappa_{b}$ is background dielectric permittivity, which describes the contribution of electronic polarization to the total electric polarization.^[42-43]^ The total electric field $E_{i}$ is the sum of the applied electric field $E_{i}^{\mathrm{ext}}$($E_{i}^{\mathrm{ext}}=0$ in this work) and the depolarization field $E_{i}^{d}$. The $E_{i}^{d}$ is obtained by numerically solving the following electrostatic equilibrium equation:

$\nabla\cdot D_{i}=\nabla\cdot(\kappa_{0}\kappa_{b}E_{i}^{d}+P_{i})=0$, (S9)

where $D_{i}$ is the electric displacement field.

The Fourier Spectral Iterative Perturbation (FSIP) method, which allows the model to include a phase-dependent (spatially inhomogeneous) background dielectric permittivity $\kappa_{b}$,^[44]^ was used to solve **equation S9**. Equation S9 also requires continuity of *D*_z_ across the PTO/STO interface. Increasing the $\kappa_{b}$ of the STO layer therefore enables more complete screening of the polarization bound charge in PTO. This treatment is also consistent with the fact that photocarriers in STO result in a significant increase in the dielectric constant.^[45]^ A similar strategy of increasing the $\kappa_{b}$ to tune the depolarization field has also been adopted in previous simulations of the topological polar phase evolution followed by above-bandgap excitation.^[46]^

The last term in the integrand of **Equation S5** is the elastic energy density:

$f_{\mathrm{elas}}=\frac{1}{2}c_{ijkl}\left( \varepsilon_{ij}-\varepsilon_{ij}^{0} \right)\left( \varepsilon_{kl}-\varepsilon_{kl}^{0} \right)$, (S10)

where $c_{ijkl}$ is the elastic stiffness tensor at constant electric field and temperature of the paraelectric PbTiO_3_ or SrTiO_3_. $\boldsymbol{\varepsilon}^{0}$ is the spontaneous strain and related to the polarization by the electrostrictive tensor $Q_{ijkl}$ via $\varepsilon_{ii}^{0}=Q_{11}P_{i}^{2}+Q_{12}(P_{j}^{2}+P_{k}^{2})$ and $\varepsilon_{ij}^{0}=Q_{44}P_{i}P_{j}$ (for $i\neq j$). The total strain **ε** can be written as $\boldsymbol{\varepsilon}=\boldsymbol{\varepsilon}^{\mathrm{eq}}+\Delta\boldsymbol{\varepsilon}\left( t \right)$. Here, $\boldsymbol{\varepsilon}^{\mathrm{eq}}$ is the total strain at the initial equilibrium state and can be obtained by numerically solving the following mechanical equilibrium equation in the framework of Khachaturyan’s mesoscopic elasticity theory:

$\nabla\cdot c_{ijkl}\left( \varepsilon_{kl}-\varepsilon_{kl}^{0} \right)=\nabla\cdot c_{ijkl}\left( \bar{\varepsilon_{kl}}+\varepsilon_{kl}^{\mathrm{het}}-\varepsilon_{kl}^{0} \right)=0$ (S11)

where $\bar{\varepsilon_{kl}}$ is the volumetric average of the total strain $\varepsilon_{kl}$; $\varepsilon_{kl}^{\mathrm{het}}$ is the heterogeneous strain with a volumetric average of zero; $\bar{\varepsilon_{kl}}$ is determined by the mechanical boundary condition.

The coherently strained PTO/STO SL on an STO substrate has $\bar{\varepsilon_{xx}^{L}}=\frac{a^{STO,s}-a^{L}}{a^{\mathrm{Ref}}}$, $\bar{\varepsilon_{yy}^{L}}=\frac{b^{STO,s}-b^{L}}{b^{\mathrm{Ref}}}$, $\bar{\varepsilon_{zz}^{L}}=-\frac{c_{12}^{L}}{c_{11}^{L}}(\bar{\varepsilon_{xx}^{L}}+\bar{\varepsilon_{yy}^{L}})$, and $\bar{\varepsilon_{yz}^{L}}=\bar{\varepsilon_{xz}^{L}}=\bar{\varepsilon_{xy}^{L}}=0$ in the PTO and STO layer (i.e., the superscript “L” indicates either the PTO or STO layer). Here $a^{STO,s}=b^{STO,s}$ are the in-plane lattice parameters (along [100]_c_ and [010]_c_, respectively) of the (001)_c_ STO substrate. $a^{L}$=$b^{L}$ are the in-plane lattice parameters (along [100]_c_ and [010]_c_, respectively) of the paraelectric (001)_c_ PTO or (001)_c_ STO layer. $a^{\mathrm{Ref}}=\frac{1}{2}(a^{\mathrm{PTO}}+a^{\mathrm{STO}})$ and $b^{\mathrm{Ref}}=\frac{1}{2}(b^{\mathrm{PTO}}+b^{\mathrm{STO}})$ are the reference in-plane lattice parameters. With the $\bar{\varepsilon_{kl}}$ and $\varepsilon_{kl}^{0}$, $\varepsilon_{kl}^{\mathrm{het}}$ is then solved numerically using the FSIP method which also allows for incorporating phase-dependent elastic stiffness coefficient $c_{ijkl}$.^[44]^

The dynamical strain Δ*ε*, which originates from the oscillating polarization, can be obtained by solving the elastodynamic equation

$\rho\frac{\partial^{2}\Delta\mathbf{u}}{\partial t^{2}}=\nabla\cdot(\Delta\boldsymbol{\sigma}+\beta\frac{\partial\Delta\boldsymbol{\sigma}}{\partial t})$ (S12)

where *ρ* is the mass density and *β* is the stiffness damping coefficient and $\Delta\mathbf{u}\boldsymbol{=}\mathbf{u}\boldsymbol{-}\mathbf{u}^{\mathrm{eq}}$ is the dynamical displacement.$\Delta\boldsymbol{\sigma}=\boldsymbol{\sigma}\boldsymbol{-}\boldsymbol{\sigma}^{\mathrm{eq}}$ is the dynamical stress, which can be also written as $\Delta\sigma_{ij}=C_{ijkl}(\Delta\varepsilon_{kl}-\Delta\varepsilon_{kl}^{0})$, with $\Delta\varepsilon_{kl}^{0}=\varepsilon_{kl}^{0}-\varepsilon_{kl}^{0, \mathrm{eq}}$ and $\Delta\varepsilon_{kl}=\frac{1}{2}(\frac{\partial\Delta u_{k}}{\partial l}+\frac{\partial\Delta u_{l}}{\partial k})$.

| **Parameter** | **PTO** | **STO** | **SRO** |
| --- | --- | --- | --- |
| ***α*_1_ (10^5^ C^-2^m^2^N)** | **3.8(*T*-752) ^[41]^** | **7.06(*T*-35.5) ^[41]^** | **/** |
| ***α*_11_ (10^8^ C^-2^m^2^N)** | **-0.73 ^[41]^** | **17.0 ^[41]^** | **/** |
| ***α*_12_ (10^8^ C^-2^m^2^N)** | **7.5 ^[41]^** | **13.7 ^[41]^** | **/** |
| ***α*_111_ (10^8^ C^-2^m^2^N)** | **2.6 ^[41]^** | **0 ^[41]^** | **/** |
| ***α*_112_(10^8^ C^-2^ m^2^ N)** | **6.1 ^[41]^** | **0 ^[41]^** | **/** |
| ***α*_123_ (10^8^ C^-2^ m^2^ N)** | **-37 ^[41]^** | **0 ^[41]^** | **/** |
| ***Q*_11_ (C^-2^ m^4^)** | **0.089 ^[41]^** | **0.0509 ^[41]^** | **/** |
| ***Q*_12_ (C^-2^ m^4^)** | **-0.026 ^[41]^** | **-0.015 ^[41]^** | **/** |
| ***Q*_44_ (C^-2^ m^4^)** | **0.0675 ^[41]^** | **0.01065 ^[41]^** | **/** |
| ***c*_11_ (10^11^ m^-2^ N)** | **1.746 ^[41]^** | **3.156 ^[41]^** | **1.07 ^[47]^** |
| ***c*_12_ (10^11^ m^-2^ N)** | **0.7937 ^[41]^** | **1.01 ^[41]^** | **0.61 ^[47]^** |
| ***c*_44_ (10^11^ m^-2^ N)** | **1.111 ^[41]^** | **1.19 ^[41]^** | **0.28 ^[47]^** |
| ***G*_11_ (10^-10^ C^-2^ m^4^ N)** | **1.038 ^[40]^** | **1.038 ^[40]^** | **/** |
| ***κ*_b_** | **40 ^[48]^** | **40 ^[48]^** | **10^3^** |
| ***μ* (J m s^2^ C^-2^)** | **6.27×10^-18^ ^[28]^** | | **/** |
| ***γ* (Ω m)** | **2.0×10^-5^, 2.0×10^-7^ ^[28]^** | | **/** |

**Table S3.** Material parameters of PbTiO_3_, SrTiO_3_ and SrRuO_3_, expressed for temperature *T* in K, with references to literature sources of the values. The notation “/” indicates quantities that are not applicable for SrRuO_3_.

Periodic boundary conditions were applied at the four lateral surfaces in the *xy* plane. The Neumann boundary condition ${\partial P_{i}}/{\partial z}=0$ was applied to the SL surface and to the SL/SRO interface.^[49]^ Along the *z*-axis, mechanical displacement and stress continuity boundary conditions were applied at the interfaces. The stress-free condition at the surface of the SL was automatically accounted for by setting the $c_{ijkl}$ to be zero in the air region. An absorbing boundary condition for the mechanical displacement, $\frac{\partial u_{i}}{\partial z}=-\frac{1}{v}\frac{\partial u_{i}}{\partial t}$ (*i* = *x*, *y*, *z*) was applied to the bottom surface of the STO substrate to suppress the reflection of the elastic wave, and thereby suppress the influence of the reflected elastic wave on the polarization dynamics in the SL. Here *v* is the transverse sound velocity for $u_{x}$ and $u_{y}$, and the longitudinal sound velocity for $u_{z}$.

The equilibrium ferroelectric domain structure was obtained by setting up a polarization distribution with random directions and small magnitude (|**P**|~0.02), simulating the paraelectric phase at high temperature, and then evolve this random polarization distribution to equilibrium at room temperature (298 K) by numerically solving **Equation** **S4, S9**, and **S11** in a coupled fashion using the methods described above. The background relative dielectric constants $\kappa_{b}$ of the STO substrate, SRO electrode, superlattice, and air layer were set to 40, 10^3^, 40, and 1, respectively. The equilibration process employed values of the mass coefficient (*μ* = 1.59 × 10^-12^ J m s^2^ C^-2^) and the damping coefficient (γ = 0.2 Ω m) that were much larger than the values given in **Table S3** in order to suppress polarization oscillations and enable faster evolution to thermodynamic equilibrium. To simulate the coupled polarization and strain dynamics in the PTO/STO superlattice after the optically induced screening of the polarization at the PTO/STO interface, we increased the $\kappa_{b}$ of the STO layer from 40 to 10^4^ at *t* = 0 ps and the numerically solved **Equation** S4, S9, and **S12** in a coupled fashion, using the equilibrated domain structure as the input. In this step, the values of the mass coefficient *μ* and damping in Table S3 were used. Both the real-space and Fourier-space numerical solvers were accelerated through graphics processing unit (GPU) parallelization.

***Calculation of X-ray diffraction patterns from the dynamical phase field simulation results***

The strain and polarization as a function of time were obtained for a SL with 120 cells along the x-direction and 110 cells along the z-direction, corresponding to 10 repeating SL units of PTO_8_/STO_3_ SL. The initial atomic positions were set such that the Ti cation displacement produced an ionic polarization state where STO layers have 40% of the polarization of the PTO layers, similar to the previous study.^[43, 50]^ The time-dependent strain determined in the simulations was added to the initial atomic positions to get a time series of atomic positions for each time step. The additional contribution to the dynamical position of the Ti cation was calculated by employing the fractional polarization ratio *P*_z_(*t*)/*P*_z_(*t* = 0). A similar approach employing atomic displacements associated with ferroelectric soft modes has been employed to connect the polarization predicted by phase field models to atomic coordinates.^[51]^ The dynamical phase field model does not consider the substrate constraint of fixed in-plane lattice parameters for epitaxial films. So, the additional contribution of out-of-plane strain (*ε*_zz_) resulting from the in-plane strain (*ε*_xx_ and *ε*_yy_) was calculated using, $\varepsilon_{zz}=\frac{2\nu}{1-\nu}(\varepsilon_{xx}+\varepsilon_{yy})$, where *ν* is the Poisson ratio.

The time-dependent atomic positions were used to calculate the scattering factor:

$F \left( Q_{z},t \right)=\sum_{k=1}^{n} f_{j}e^{-i\vec{Q}_{z}\cdot\vec{r}_{j,t}}$, (S13)

where *n* is the number of atoms, *f*_j_ is the atomic scatting factor of atom *j*, *Q*_z_ is the scattering vector and *r*_j,t_ is the position vector of atom *j* at time *t*. The X-ray intensity distribution as a function of wavevector and time was obtained computing the square magnitude of the scattering factor $I\left( Q_{z},t \right)=\left| F\left( Q_{z},t \right) \right|^{2}$.

The intensity distribution calculated for PTO_8_/STO_3_ SL calculated from the dynamical phase field simulation result is shown in **Figure S7**. The CWT of the intensity distribution shown in Figure S7 is shown in Figure 4d.


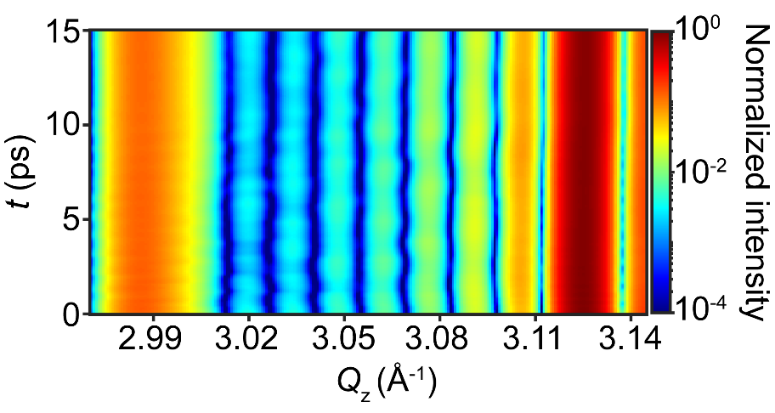


**Figure S7. Simulated X-ray diffraction intensity.** Diffracted X-ray intensity as a function of *Q*_z_ and *t* for PTO_8_/STO_3_ SL calculated from the dynamical phase field simulation results.

Other PTO/STO SLs with different STO fractions were also studied in the dynamical phase field calculations. The calculated X-ray intensity distribution for PTO_6_/STO_4_, PTO_5_/STO_5,_ and PTO_4_/STO_6_ SLs and their corresponding time-averaged CWT amplitudes are also shown in **Figure S8**. The frequency mode observed corresponding to the dynamical transition in polar texture was determined using Figure S8 and reported in the main text.


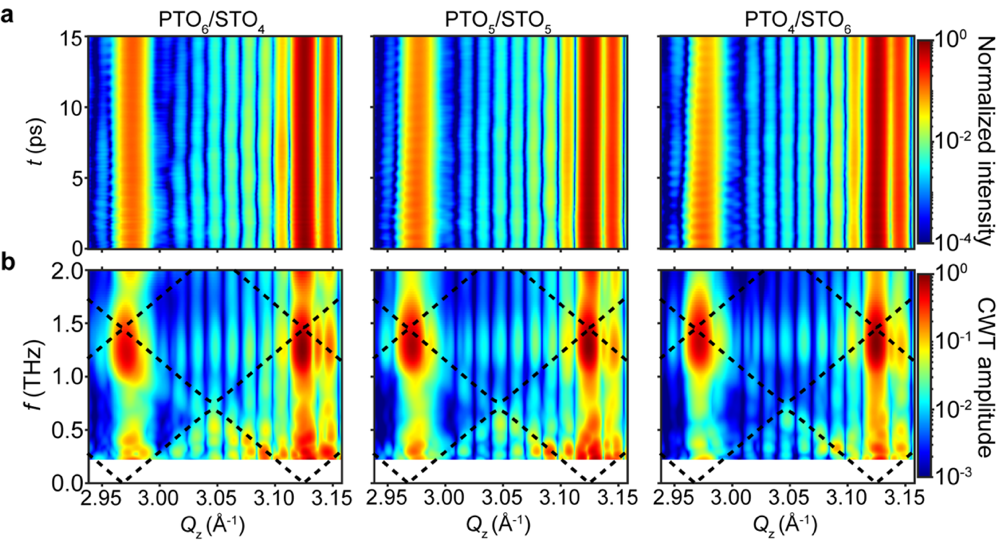


**Figure S8. Simulation results for additional superlattice component layer thicknesses.** a) Diffracted X-ray intensity as a function of *Q*_z_ and *t* for PTO_6_/STO_4_, PTO_5_/STO_5_ and PTO_4_/STO_6_ SLs calculated from the dynamical phase field simulation results. b) Time-averaged CWT amplitude computed from the intensity maps in in b.

***Optical-Pump-Off Measurements***

The measurement included randomly interleaved x-ray pulses for which the optical pump was turned off. The unpumped shots provided a baseline to investigate possible domain transformations or effects arising from cumulative optical exposure. The diffuse scattering intensity around the SL *l*=0 reflection for the optical-pump-off pulses as a function of delay time is shown in Fig. S9. There is no variation in the unpumped intensity throughout the experiment. We thus conclude that the polarization configuration relaxes back to the same initial polarization configuration before each pulse and that no metastable domain configurations are populated under these photoexcitation conditions.

***
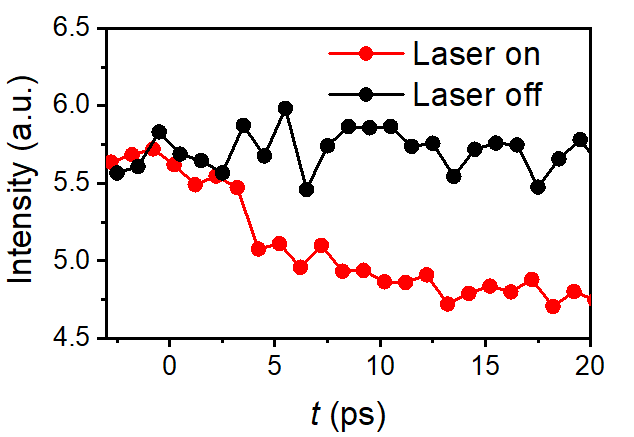
***

**Figure S9.** Time-dependent intensity of domain diffuse scattering around SL *l*=0 reflection in the presence (laser on) and absence (laser off) of the optical pump pulses.
